# Antibacterial and Cytotoxic Activities of Ten Commercially Available Essential Oils

**DOI:** 10.3390/antibiotics9100717

**Published:** 2020-10-20

**Authors:** Sofia Oliveira Ribeiro, Véronique Fontaine, Véronique Mathieu, Abdesselam Zhiri, Dominique Baudoux, Caroline Stévigny, Florence Souard

**Affiliations:** 1 Department of Research in Drug Development (RD3), Pharmacognosy, Bioanalysis and Drug Discovery Unit, Faculty of Pharmacy, Université libre de Bruxelles, Boulevard du Triomphe, 1050 Brussels, Belgium; Caroline.Stevigny@ulb.be; 2 Department of Research in Drug Development (RD3), Microbiology, Bioorganic and Macromolecular Chemistry Unit, Faculty of Pharmacy, Université libre de Bruxelles (ULB), Boulevard du Triomphe, 1050 Brussels, Belgium; veronique.fontaine@ulb.be; 3 Department of Pharmacotherapy and Pharmaceutics, Université libre de Bruxelles (ULB), Boulevard du Triomphe, 1050 Brussels, Belgium; Veronique.Mathieu@ulb.ac.be; 4 Pranarôm International S.A. 37, Avenue des Artisans, 7822 Ghislenghien, Belgium; abdesselam.zhiri@ulb.ac.be (A.Z.); dbaudoux@pranarom.com (D.B.); 5 Unité de Recherche en Biotechnologie Végétale, Université libre de Bruxelles, CP 300, Rue Prof. Jeener & Brachet 12, 6041 Gosselies, Belgium; 6 Department of Pharmacotherapy and Pharmaceutics (DPP), Pharmacology, Pharmacotherapy and Pharmaceutical care Unit, Faculty of Pharmacy, Université libre de Bruxelles (ULB), Boulevard du Triomphe, 1050 Brussels, Belgium; Florence.Souard@ulb.be; 7 Département de Pharmacochimie Moléculaire (DPM), Université Grenoble Alpes, CNRS, UMR 5063, F3Y041 Grenoble, France

**Keywords:** essential oil, bacterial infections, bacterial resistance, antibacterial activity, MRSA, cytotoxicity

## Abstract

There is a huge concern in the medical field concerning the emergence of bacterial resistance to antibiotics. Essential oils are a source of antibacterial compounds that can overcome this problem. Ten essential oils that are commercially available were investigated in the present study: ajowan, basil, German chamomile, Chinese cinnamon, coriander, clove, lemongrass, Spanish lavender, oregano and palmarosa. Their direct, synergistic and indirect antibacterial activities were evaluated against different human pathogenic Gram-positive and Gram-negative strains. To evaluate their possible use in clinics, the cytotoxicity of these essential oils was also tested on keratinocyte and epithelial cell lines. Except for the Chinese cinnamon, coriander and lemongrass, all other essential oils presented no cytotoxicity at 32 and 16 μg/mL. The highest indirect antibacterial activities were observed with the palmarosa and Spanish lavender in association with penicillin V. These two associations presented a 64-fold decrease against a resistant strain of *Staphylococcus aureus*, however, at a cytotoxic concentration. It can also be highlighted that when tested at a non-cytotoxic concentration, the activity of oregano in association with penicillin V presented an eight-fold decrease. These results show the interest to use essential oils in combination with antibiotics to reduce their concentrations inside drugs.

## 1. Introduction

The bacterial resistance to antibiotics remains a huge threat to public health and is still associated with high mortality and morbidity [1]. The World Health Organization (WHO) published a list of priority pathogens (from medium to critical level) to be targeted for antimicrobial research [2] and, worldwide, governments and organizations have published reports or global action plans to tackle this health problem [1,3,4]. Despite the awareness of this global situation, the list of resistant pathogenic strains has continued to increase over the last twenty years, with a resistance now extended to more than one family of antibiotics [5]. To address this increase in bacterial resistance, it is necessary to have better management of the antibiotics consumption in both human and veterinary health, but also to promote scientific research to find new molecules with antibacterial activity that can inhibit the bacterial resistance and/or work in synergy with antibiotics [6,7]. Regarding this last point, researchers have been looking for new molecules coming from plant material [8,9], with a special focus on essential oils. Essential oils are naturally produced by different organs in aromatic plants and possess a complex chemical composition that can be a potential source of new antibacterial compounds [10,11]. The aim of this work was to evaluate both antibacterial (direct, indirect and synergistic) and cytotoxic activities of ten commercial essential oils (ajowan—*Trachyspermum ammi* L., basil—*Ocimum basilicum ssp basilicum* L., German chamomile—*Matricaria recutita* L., Chinese cinnamon—*Cinnamomum cassia* (L.) J. Presl., coriander—*Coriandrum sativum* L., clove—*Eugenia caryophyllus (Spreng.) Bullock and S. G. Harrison*, lemongrass—*Cymbopogon citratus* (DC.) Staph., Spanish lavender—*Lavandula stoechas* L., oregano—*Origanum compactum* Benth., and palmarosa—*Cymbopogon martinii var. motia* (Roxb.) W.Watson.

To contribute further to the fight against the increased resistance of pathogenic bacteria, we specially focused our study on the indirect activity of essential oils, while most of other studies focused on synergy assays. Both assays allow one to evaluate the synergy activities of the association between essential oils and antibiotics (on a resistant strain). However, in the indirect test, the essential oil is tested at a fixed non active concentration (two dilutions below the direct MIC = sub-MIC = sub minimum inhibitory concentration) in association with an antibiotic. The objective is to enhance or restore the activity of the antibiotic (on a resistant strain) without introducing a new active compound in the resistance equation. In our protocol, this sub-MIC concentration has been fixed at a non-cytotoxic concentration to obtain results that could allow further exploitations. To do so, the cytotoxicity in vitro of all the essential oils was also tested against a keratinocyte and an epithelial cell line to check the possibility of using them either topically or orally. Finally, in this manuscript, we best tried to correctly describe all the methods and the results, allowing an adequate comparison with other previously published studies that possibly used different parameters in the test methodology, different strains from other collections or essential oils with different chemical composition.

## 2. Results

### 2.1. Essential Oil’s Cytotoxic Activity

To reduce the toxicity risk towards human healthy cells at the site of use, we first evaluated the possible cytotoxic/anti-proliferative activities of essential oils towards a human skin keratinocyte cell line (HaCaT) and a human fetal epithelial colon cell line (CoN). A residual cell (RC) viability of 80%, after 72 h of culture, was considered as a minimum requirement to express the possible local tolerance, i.e., when essential oils are used in a topic or an oral way of administration for veterinary or human purposes. The detailed results are presented in Table 1. Only Chinese cinnamon showed toxicity at all the tested concentrations on both cell lines. On the HaCaT keratinocytes cell line, lemongrass also presented cytotoxicity at all the tested concentrations and, oppositely, basil showed no cytotoxicity at all. The ajowan and palmarosa showed no cytotoxicity at concentrations ≤ 125 μg/mL and all the other essential oils presented no cytotoxicity at 32 and 16 μg/mL. On the CoN epithelial cell line, palmarosa showed no cytotoxicity at all the tested concentrations, whereas basil, German chamomile and clove only showed cytotoxicity at the highest concentration of 250 μg/mL. The ajowan, lemongrass, Spanish lavender and oregano are not cytotoxic at 32 and 16 μg/mL, and coriander is not cytotoxic at 16 μg/mL only.

### 2.2. Essential Oil’s Direct Activity

All essential oils were tested for their direct antibacterial activity against nine strains: five Gram-negative strains, i.e., *E. coli* LMG 8223, *E. coli* LMG 15862, *P. aeruginosa* LMG 6395, *K. pneumoniae* LMG 20218 and *E. aerogenes* LMG 2094 and four Gram-positive strains, i.e., *S. aureus* LMG 8064 (MSSA), *S. aureus* LMG 15975 (MRSA), *S. aureus* LMG 16217 (MRSA) and *E. faecalis* LMG 8222. The results observed against the Gram-negative bacteria (Table 2) showed that Chinese cinnamon and oregano are the more active with MIC ranging from 250 to 500 μg/mL. Against *E. coli* LMG 8223, *K. pneumoniae* and *E. aerogenes*, an MIC of 500 μg/mL was also observed with ajowan.

The results obtained for the Gram-positive strains are detailed in Table 3. When testing the MIC of the different antibiotics used in synergy and with indirect activities, we observed that *S. aureus* LMG 16217 is resistant to more antibiotics than *S. aureus* LMG 15975. Only Chinese cinnamon showed an effect against *E. faecalis* with an MIC of 500 μg/mL. Against the three *Staphylococci* strains, coriander was active at 125 μg/mL, Chinese cinnamon and oregano showed an MIC at 250 μg/mL and ajowan was less active with an MIC at 500 μg/mL. The essential oil of lemongrass was active at 250 μg/mL against the MSSA and the more resistant *S. aureus* LMG 16217 while palmarosa showed only activity against the MSSA with an MIC at 500 μg/mL.

### 2.3. Synergistic Activity between Essential Oils and Antibiotics

The classic synergistic activity was evaluated for the ten essential oils in association with different antibiotics against the two MRSA and the two Gram-negative, *E. coli* LMG 15862 and *P. aeruginosa* LMG 6395. Those strains were chosen because they present resistance to most of the tested antibiotics and are among the most known and studied human pathogenic strains. The associated clinically used antibiotics, generally administrated orally, were chosen because the related strains are resistant to them as seen in Table 2 and Table 3. For this assay, we combined one essential oil and one antibiotic serially diluted from 1000 μg/mL and 64 μg/mL, respectively, in a 1:1 ratio. The association (synergy, additive, indifferent or antagonist) was determined according to the FIC index (Fractional Inhibitory Concentration Index). The FIC index and the corresponding interaction can be found in Table 4. The detailed values and the FIC calculations can be found in the Supplementary Material (Table S1a–d). Regarding the results on Gram-negative strains, only six additive interactions (basil, German chamomile, coriander, lemongrass, Spanish lavender and palmarosa) were observed against *P. aeruginosa* LMG 6395 with doxycycline. By contrast, for the MRSA strains, almost all the combinations showed an additive or a synergistic effect. In particular, against the most resistant MRSA *S. aureus* LMG 16217, three synergistic associations were observed: the German chamomile and clove with penicillin V, and the lemongrass with amoxicillin. Three synergistic associations were also observed against *S. aureus* LMG 15975: the Chinese cinnamon, coriander and oregano in association with amoxicillin.

### 2.4. Essential Oil’s Indirect Activity

The results of the indirect activity tested for the ten essential oils against the Gram-negative, *E. aerogenes* and *K. pneumoniae*, are presented in Table 5. The indirect activity assay allowed us to investigate if an essential oil at a sub-MIC concentration, i.e., a non-active concentration, can inhibit the mechanism of bacterial resistance and restore or enhance the activity of an antibiotic. The sub-MIC was fixed as two dilutions under the MIC obtained for each essential oil per strain. Against the Gram-negative strains, some associations showed no indirect activity, which means that there was no change in the MIC of the antibiotic itself when comparing the MIC of the antibiotic alone and the MIC of the association (essential oils and antibiotic). This was observed against *E. coli* LMG 15862 (amoxicillin and penicillin V), *P. aeruginosa* LMG 6395 (doxycycline, tetracycline and cefotaxime), *E. faecalis* LMG 8222 (cefotaxime) and *K. pneumoniae* LMG 20218 (penicillin V and ampicillin). The other associations which presented an indirect activity against Gram-negative strains are detailed in Table 5. Against *K. pneumoniae*, the association of cefotaxime–Spanish lavender decreased four-fold the MIC of the cefotaxime when used alone (from 32 to 8 μg/mL) and against *E. aerogenes*, the association of cefotaxime–oregano also decreased by four-fold the MIC of cefotaxime when used alone (from 4 to 1 μg/mL).

The same assay was also performed on Gram-positive strains. The results are detailed in Table 6. The best associations were observed against *S. aureus* LMG 15975 with the palmarosa and Spanish lavender in association with penicillin V: the MIC of penicillin V decreased from 4 μg/mL (alone) to 0.06 μg/mL (in association), which corresponds to a 64-fold decrease. Other interesting decreases in the MIC of penicillin V were observed: with the clove and German chamomile (32-fold), with the oregano and lemongrass (16-fold), with the ajowan (8-fold) and with the Chinese cinnamon (4-fold). Against the same strain, amoxicillin was decreased by eight-fold in association with the ajowan, clove and Spanish lavender and by four-fold in association with the other seven essential oils. Against *E. faecalis*, an indirect activity was observed with gentamicin when associated with the ajowan, German chamomile and lemongrass (64-fold), with the coriander and oregano (32-fold), with the clove and palmarosa (16-fold) and with the Chinese cinnamon and Spanish lavender (8-fold). Some associations of the essential oils with fosfomycin against *E. faecalis* on the one hand and with amoxicillin and penicillin V against *S. aureus* LMG 16217 on the other hand showed a two-fold decrease. These latest associations were not further investigated since the results were considered as not significant.

Nevertheless, to analyze these results more precisely, the cytotoxicity of the essential oils should be considered. Regarding the results obtained in cytotoxicity on the keratinocyte and the epithelial cell lines, seven essential oils were tested at a sub-MIC of 32 μg/mL in association with penicillin V against MRSA LMG 15975, as a model. Even when the sub-MIC is decreased, the German chamomile, clove, Spanish lavender, oregano and palmarosa still have an indirect activity. The oregano decreased the MIC of penicillin V by eight-fold and the other decreased the activity by four-fold.

### 2.5. Direct and Indirect Activities of Each Major Compound of the Essential Oils

The chemical composition of the main compounds (present at more than 10%) of each studied essential oil was obtained by GC–MS. To complete this analysis of the main compounds, the complete chemical profiles furnished by the manufacturer can be found in the Supplementary Material (Figures S1–S10). The relative abundance of each product obtained by our GC–MS analyses (Table 7), expressed in percentages, is in agreement with the data furnished by the manufacturer.

In order to better rationalize if the activity of essential oils is correlated with their chemical compositions, the major compound of each essential oil was tested alone (direct activity) and in association with penicillin V (indirect activity) against *Staphylococcus aureus* LMG 15975. Those particular strains and antibiotics were selected according to our previous results, because they appear to be the most promising combinations when testing the indirect activity (Table 6), which was our special focus in this work. All essential oils presented an indirect activity in association with penicillin V against *S. aureus* LMG 15975, decreasing the MIC of the penicillin V by 2-fold (not significantly for basil or coriander), 4-fold (for Chinese cinnamon), 8-fold (for ajowan), 16-fold (for lemongrass and oregano), 32-fold (for German chamomile and clove) and in a surprising way even by 67-fold (for Spanish lavender and palmarosa). In a complementary way, for coriander, the second majority compound was tested since its first one, linalool, did not show direct activity. Therefore, to correctly interpret the microbiological activities of essential oils, we pointed out the importance of considering all MICs in relation with the relative abundance of the different compounds (the first major at least). The results are resumed in Table 8.

Regarding the direct activity, the major compounds of Chinese cinnamon (E-cinnamaldehyde) and of lemongrass (citral) were both identified as the main responsible factors for the direct activity of these essential oils against the strain *S. aureus* LMG 15975. On the one hand, the MICs were similar between the essential oils and their major compound, and on the other hand, the percentages (82.05% and 75.16%, respectively) of these compounds in the essential oils were significant enough to be correlated to the activity. The thymol present at 45.17% in ajowan and the carvacrol present at 57.21% in oregano can also be correlated to their activity since their MIC alone are twice the one found for their respective essential oils and they represent half of the composition. The 2-decenal present at 11.07% in coriander is not concentrated enough to be responsible for the entire direct activity of this essential oil. The slight activity of the basil and palmarosa can be attributed to their major compounds, estragole (87.58%) and geraniol (81.05%), respectively.

To analyze the indirect activity of these major compounds when they are associated with penicillin V, it is essential to compare it to the indirect activity of the essential oils associated with the same antibiotic, and to take into account both the percentage of the compound in the essential oil and the tested sub-MIC. Three different groups can be observed. In the first group, the major compounds could be identified as mainly responsible for the activity of their respective essential oils. The estragole (present at 87.58% in basil) presents the same indirect activity at the same tested sub-MIC and similarly we can also identify the citral present at 75.16% in lemongrass and the geraniol present at 81.05% in palmarosa.

In the second group, the indirect activity of the essential oil does not appear to be attributed to its major compound. Indeed, the MIC of the major compound did not reach the one obtained for the essential oil at the same sub-MIC. This was observed for the Chinese cinnamon composed at 82.05% of E-cinnamaldehyde and for the clove composed at 84.58% of eugenol. It means that the compound(s) responsible for the indirect activity of the Chinese cinnamon and clove is/are present in less than 20%.

In the third group, the major compound appeared to be one of the compounds responsible for the activity of the respective essential oil. At a sub-MIC of 125 μg/mL, the ajowan decreased the MIC of penicillin V by eight-fold, while its major compound, thymol (45.17%), decreased it by 32-fold with a lower sub-MIC (64 μg/mL). This difference may indicate antagonist compound(s) acting on thymol activity. On the contrary, synergism can be identified for two essential oils. The oregano decreased by 16-fold the MIC of penicillin V at a sub-MIC of 64 μg/mL, while carvacrol (present at 57.21%), tested at a sub-MIC of 32 μg/mL, decreased it by eight-fold. A similar result was observed for the Spanish lavender composed at 32.54% by camphor, which presented a lesser indirect activity (eight-fold) than the essential oil (67-fold) at the same sub-MIC. These differences could indicate that these major compounds act in synergy with one or more compounds.

For the last two compounds (E-β-farnesene and 2-decenal), their percentages seem to be not sufficient enough to be responsible for the entire activity of their respective essential oil, because: E-β-farnesene is present at 34.61% in German Chamomile and 2-decenal at 11.07% in coriander only.

## 3. Discussion

Essential oils are known to be anti-infectious [11]. Clinically, it is common to use a combination of antibiotics to eradicate multi-drug resistant bacteria. With this work, an alternative combining both of these properties is presented: the use of an essential oil combined with an antibiotic. Ten different essential oils were investigated for their antibacterial activity (direct activity) against commonly human pathogens, for their ability to act in synergy with antibiotics (synergistic activity) and/or to inhibit the resistant mechanisms of bacteria and allow the restoring of the antibiotic’s activity (indirect activity). In order to hypothetically correlate and understand the mode of action of the essential oils, the activities of their major compounds were also studied. The choice of the antibiotics was based on their clinical way of administration, either orally or topically. A preliminary safety evaluation was carried out by testing the essential oils at different concentrations on common models of keratinocyte and epithelial cell lines.

Different parameters were taken into account during the planification of this study. The first one was to test the essential oils by the microdilution method. In fact, the MIC obtained by the agar disk diffusion method and by the broth dilution method can give variable results [10,12]. In addition, as mentioned by different authors including Becerril et al. (2012) [13] and Orchard and Van Vuuren (2017) [10], it is not accurate to compare two MICs obtained from two different strains. In this paper, the resistance of the tested strains was checked by comparing the MIC obtained with the European Committee on Antimicrobial Susceptibility Testing (EUCAST) breakpoints tables [14], and to be more accurate, whenever possible, the obtained results were compared with papers which tested the same reference strain. Another crucial point to take into consideration when testing essential oils is their variable chemical composition, which can be influenced by many factors such as the genotype, the origin, the part of the plant used and/or the harvest time [15]. Another important point is the correlation between antibacterial activity and cytotoxicity. Many authors decide to test the cytotoxic activity of essential oils against cancer cell lines. The results of such biological activities should always be related to their cytotoxic activity on healthy cell lines [16]. For further development, it is important that the essential oils have a selective action. In our study, we fixed a cut off limit in the cell viability (80%) for further development. It is important to notice that at the lowest concentrations (32 and 16 μg/mL) all the tested essential oils, except the Chinese cinnamon and lemongrass, were non-toxic on the keratinocyte cell line (HaCaT). Similar results were observed on the epithelial cell line (CoN), except for the Chinese cinnamon and coriander. The results discussed below for these three essential oils should, therefore, be carefully interpreted.

Regarding the antibacterial activity, from a general point of view, essential oils were more active against the tested Gram-positive than the Gram-negative strains. Given the structural difference of the membranes between Gram-positive and Gram-negative bacteria, this observation is not surprising and has been countlessly reported in the literature [10,17,18]. The cell envelope of Gram-positive strains is composed of glycopolymers such as teichoic acid and several layers of peptidoglycan, making the envelope thicker than the one of Gram-negative strains, which is composed of a thin layer of peptidoglycan [19,20]. However, Gram-negative strains are naturally more resistant due to an outer membrane composed of lipoproteins and lipopolysaccharides, which reduces the entrance of certain drugs such as lipophilic molecules [18,19,20]. Given their hydrophobicity, it may be hypothesized that the essential oils are, a priori, more effective against Gram-positive strains since they can more easily penetrate and accumulate in the phospholipidic layer causing conformation changes and dysregulations in the cell membrane [21]. Still, the outer membrane does not confer a complete impermeability to Gram-negative [18]. On the one hand, lipophilic compounds can, even if they are highly retarded, pass through the porins, that normally are present to allow the passage of hydrophilic compounds [19]; but on the other hand, they can also affect the percentage of unsaturated fatty acids and their structure, leading to an alteration of the membrane structure and therefore to an increase in the permeability of the outer membrane [18]. So, even if most of hydrophobic compounds, like those present in essential oils, are generally more active against the Gram-positive, they still have some activity against the Gram-negative. In line with this affirmation, we observed in our study that the Chinese cinnamon and oregano showed direct activity on both Gram-negative and Gram-positive tested strains, and the ajowan did not show activity on *P. aeruginosa* only. According to the literature, essential oils exert their activities through different mechanisms, such as membrane destabilization or different inhibition such as ATPase activity, cell division or efflux pumps activity [18,21]. The activity of essential oils depends on their chemical composition, but most of the time studies indicate the membrane as the first target independently of the active compound or the bacteria strain [18,21]. The E-cinnamaldehyde present at 82.05% in Chinese cinnamon, thymol present at 45.17% in ajowan and carvacrol present at 57.21% in oregano are not an exception: most of the studies indicate the membrane integrity as the first target [17]. Those three compounds can at certain doses perturb in vitro the lipid membrane of the bacteria causing changes in the permeability, causing the leakage of the intracellular material and therefore the death of the bacteria [17,18,22,23]. However, studies have demonstrated that E-cinnamaldehyde, for example, can also act via other mechanisms such as the inhibition of the protein FtsZ, a protein responsible of the cell division, or the inhibition of ATPase, causing a decrease in transportation by reduction in ATP or pH regulation [18,24]. As pointed by Memar et al. [23], knowing the mechanism of action of the essential oils is one thing, but to achieve a clinical use, the research of the toxicity should not be forgotten. E-cinnamaldehyde is known to present toxicity against cancer cell lines but also against normal cell lines, as observed in the present study [25,26]. As mentioned before, the results regarding Chinese cinnamon should be carefully interpreted. To our knowledge, this is the first description of ajowan essential oil activity against the tested MRSA strains, LMG 15975 and 16217, although previous literature already mentioned an antibacterial activity against *E. coli* LMG 8223 [27,28], *P. aeruginosa* LMG 6395 [28,29,30] and MSSA LMG 8064 [31], as observed in the present study. For the Chinese cinnamon, who is cytotoxic in both tested cell lines, our results presented a better MIC than the one described by Kaskatepe et al. (2016) [32] against the same strain of *P. aeruginosa* LMG 6395 and the one described against *E. coli* LMG 8223 [33,34]. As mentioned before, oregano was active against all the tested Gram-negative strains, including *P. aeruginosa*, which is one of the ESKAPE pathogens that need to be eradicated the most [35]. According to the literature, it seems that it is the first time that oregano showed a direct activity against the strain *P. aeruginosa* LMG 6395. Indeed, Ouedrhiri et al. (2016) [36] and Bouhdid et al. (2009) [37] described an MIC superior to 10 mg mL^−1^ while we observed an MIC of 500 μg/mL. Against the same strain of *E. coli* LMG 15862, Ouedrhiri et al. (2016) [36] described an MIC similar to ours but Plant et al. (2015) [38] described a lower activity with an MIC of 1000 μg/mL. Against the Gram-positive strain MRSA LMG 15975, we observed an MIC of 250 μg/mL but Haba et al. (2014) [39] described an MIC five times superior (1250 μg/mL).

The differences observed between the authors can arise from various reasons. The two more probable ones are the use of different protocols or/and the fact that a different chemotype could be used. The chemical composition of essential oils depends on many factors whether abiotic or biotic [40]. For example, the Chinese cinnamon used by Huang et al. (2014) [33] is composed at only 69% of cinnamaldehyde and he used the macrodilution to determine the MIC, instead of the microdilution. On another hand, the Chinese cinnamon used by Zhang et al. (2016) [34] has a similar composition but he used Tween 80 (undescribed %) to dilute the essential oils, instead of the DMSO (5% in our case). Haba et al. (2014) [39] described an MIC five times superior than ours against MRSA LMG 15975 but the chemical composition of both oregano is different with only 38% of carvacrol compared to ours (57%). Due to the high variability of the essential oils, rare are those with a defined chemotype, and consequently available ways to determine the MIC. Therefore, it should be mandatory to always indicate the chemical composition of the tested samples together with a detailed protocol.

Still, regarding the direct activity on Gram-positive *S. aureus*, interestingly, the ajowan, Chinese cinnamon, coriander and oregano presented the same MIC against the sensitive strain (MSSA) and the two resistant strains (MRSA). They have an antibacterial activity independent of the level of resistance to the β-lactam. This supposedly means that their modes of action are different from the antibiotics one. Indeed the mode of action of the β-lactams, such as penicillin V, is by interfering with the peptidoglycan biosynthesis [41] while, as discussed above, the mode of action of thymol, carvacrol and cinnamaldehyde is mainly the disruption of the lipid membrane [42]. The synergy between essential oils and antibiotics were also investigated in this paper aiming to reduce the administrated concentration of antibiotics. It is important to point out that no antagonism was found. The German chamomile and clove can be highlighted because they did not show direct activity, but they act in synergy with penicillin V against MRSA LMG 16217, the most resistant MRSA tested. The correlation between the synergy and the chemical composition was not studied in this paper. However, it is known that the additive or synergic effect observed with most of the essential oils depends on the major compound, but more likely on the synergy between two or more compounds which can be present in minor concentrations [43].

To objectify the synergy, another perspective studied was the indirect activity where the concentrations of the tested essential oils are fixed at a sub-MIC concentration (non-active alone). With this test, we really observed the increased potency of the antibiotic; likewise, the association of ampicillin–clavulanic acid where clavulanic acid enhances the activity of ampicillin [44]. Almost all of the essential oils enhanced the activity of penicillin V against the MRSA LMG 15975. Due to the lipophilic nature of essential oils, they are able to penetrate and accumulate in the phospholipidic membrane and disrupt its regulation [21]. Even at a non-active concentration, this property to interact with the membrane is probably the reason why they can enhance the activity of the antibiotic. The difference with a synergistic activity is that the essential oil is at a non-active concentration. This may cause a delay in the resistant response of the bacteria to the antibiotic. The Spanish lavender and palmarosa presented no direct activity and only an additive effect when tested for the synergistic activity. However, at a sub-MIC of 500 and 250 μg/mL, respectively, they were able to decrease by 67-fold the MIC of penicillin V alone (4 to 0.06 μg/mL). Knowing that the MIC of penicillin V against the MSSA tested was 0.015 μg/mL, these results were particularly interesting. These associations are able to restore the activity of penicillin V on a resistant MRSA to a level close to the effective dose on a sensitive strain (MSSA). The indirect activity of palmarosa can be related to its major compound, geraniol present at 81.05%. In the case of the Spanish lavender it seems that camphor, only present at 32.54%, acts in synergy with other compound(s). The indirect activity of camphor described an eight-fold decrease in penicillin V, much less than the one observed for the entire essential oil (67-fold). Despite these good results, the indirect activity of essential oils needed to be put into perspective regarding the cytotoxicity observed on the cell lines HaCaT and CoN. In fact, the sub-MIC at which the essential oils have been tested is cytotoxic on one or both of the cell lines. The sub-MIC concentration of 32 μg/mL concentration has been further chosen for indirect antibacterial activity in association with the penicillin V against *Staphylococcus aureus* LMG 15975. This sub-MIC was chosen because seven essential oils out of the ten are not toxic at this concentration, whereas these strains and antibiotics were chosen because they showed the best results in indirect activity with most of the essential oils. The coriander, Chinese cinnamon and lemongrass were not tested since they showed cytotoxicity even at 32 μg/mL. Reducing the Sub-MIC almost by 15 times affected seriously the indirect activity, but the association of the German chamomile, clove, Spanish lavender, palmarosa and especially the oregano is still able to increase the activity of penicillin V by four- to eight-fold. Regarding the indirect activity observed for the compounds alone, it would be interesting to test their cytotoxic activities on the same cellular lines and see if they can be used at the tested sub-MIC. For example, if the 2-decenal shows no cytotoxicity at 64 μg/mL, it would be more interesting to use it alone than coriander essential oil which presents toxicity even at a low concentration. Despite this perspective, at a low concentration, seven of the ten essential oils can promote the reduction in the administered antibiotic concentrations suggesting that further study investment should be performed on these essential oils to boost the development of new drug combination in the fight against the bacterial resistance to antibiotics.

## 4. Materials and Methods

### 4.1. Plant Material

Ten essential oils were provided by Pranarôm International S.A (Ghislenghien, Belgium): AW—ajowan (*Trachyspermum ammi* L.), batch OF17650; BE—basil (*Ocimum basilicum ssp basilicum* L.), batch OF22779; CA—*Matricaria recutita* L. (German chamomile), batch OF22830; CC—Chinese cinnamon (*Cinnamomum cassia* (L.) J. Presl.), batch OF22426; CF—coriander (*Coriandrum sativum* L.), batch OF21374; GF—clove (*Eugenia caryophyllus (Spreng.) Bullock and S. G. Harrison*), batch OF22293; LG—lemongrass (*Cymbopogon citratus* (DC.) Staph.), batch OF20407; LS—Spanish lavender (*Lavandula stoechas* L.), batch OF19205; OC—oregano (*Origanum compactum* Benth.), batch OF22429; PM—palmarosa (*Cymbopogon martinii var. motia* (Roxb.) W.Watson), batch OF21237. All of the essential oils have a certificate of analysis that can be found in the Supplementary Material (Figures S1–S10). The origin and organ used can be found in Table 7.

### 4.2. Chemicals, Bacterial Strains, Cell Lines and Growth Mediums

Dimethyl sulfoxide (DMSO), all the bacterial mediums, the 3-(4,5-dimethylthiazol-2-yl)-2,5-diphenyltetrazolium bromide (MTT) and the reference compounds were purchased from Sigma-Aldrich (Saint-Louis, IL, USA). The physiological solution (NaCl 0.85%, 2 mL) was purchased from BioMérieux (Marcy-l’Étoile, France) and all the antibiotics were purchased from Alfa Aesar (Ward Hill, MA, USA). The cell growth mediums and supplements were purchased from Thermofisher (Waltham, MA, USA) except for the fetal bovine serum who was purchased from Greiner (Kremsmünster, Autriche). The keratinocyte cell line (HaCaT) and the human fetal epithelial cells from colon (CoN–CRL-1790) were purchased from Promocell (Germany) and from ATCC (USA), respectively. The strains (four Gram-positive and five Gram-negative strains) were purchased from the Belgian Coordinated Collection of Microorganisms (BCCM/LMG, Ghent, Belgium). Mueller Hinton broth (MHB) and Tryptic Soy agar (TSA) were used with the Gram-positive strains *Staphylococcus aureus* LMG 8064 (MSSA), *S. aureus* LMG 15975 (MRSA), *S. aureus* LMG 16217 (MRSA) and the Gram-negative strains *Escherichia coli* LMG 8223, *E. coli* LMG 15862, *Pseudomonas aeruginosa* LMG 6395, *Enterobacter aerogenes* LMG 2094 and *Klebsiella pneumoniae* LMG 20218. For the Gram-positive strains, *Enterococcus faecalis* LMG 8222 blood heart infusion (BHI) broth/agar was used.

### 4.3. GC–MS Analysis

The separation and identification of the main chemical compounds of each essential oil were carried out by gas chromatography (Trace GC ultra, Thermo, MN, USA), coupled to a mass spectrometer (Polaris-Q, Thermo, USA). Helium was used as carrier gas (1 mL/min) and the column used was a Rxi-5Sil MS (Restek, France) (40 m × 0.18 mm; thickness 0.18 µm). The injector temperature was 240 °C while the detector’s temperature was 200 °C. In total, 0.8 µL of the solution prepared in hexane at 0.5% was injected for each essential oil (splitless mode). The temperature program was set at 50 °C during 6 min, then increased at a rate of 2 °C/minute until reach 250 °C and held for 10 min. The essential oil components were identified based on their retention indices (comparing to the major compounds also analyzed) and peak area percents were used to obtain quantitative data (*n* = 2).

### 4.4. Essential Oil’s Cytotoxic Activity

Human keratinocytes cells (HaCaT) were grown in Roswell Park Memorial Institute (RPMI)(supplemented with 10% of Fetal Bovine Serum (FBS) and 0.5% of penicillin-streptomycin) and the human fetal epithelial cells from colon (CoN) were grown in MEM supplemented with 10% fetal bovine serum, 0.5% essentials amino acids, 0.5% pyruvate sodium and 0.5% penicillin-streptomycin). The effect of each essential oil on the global growth of the cell line was investigated through the colorimetric MTT assay [45]. The cells seeded at a density of 10,000 cells mL^−1^ and 35,000 cells/mL, for HaCaT and CoN, respectively, were first incubated at 37 °C in an 5% CO2 humidified atmosphere in a 96-microwell plate for 24 h. Then, the cells were treated with four different concentrations of the essential oils (250, 125, 32 and 16 μg/mL) and incubated during 72 h. Next, the medium was removed by turning over the microplates and replaced by 100 μL of MTT solution (0.5 mg/mL). The plates were incubated between 3 to 5 h in the cell culture incubator to allow viable cells to reduce the tetrazolium salt into formazan. The plates were centrifuged at 1200 rpm for 5 min. The supernatant was discarded, and the formazan crystals formed dissolved in 100 μL of DMSO. The microplates were shacked for 5 minutes (450 rpm) and read on a spectrophotometer (680XR, BioRad, Hercules, CA, USA) at 2 wavelengths (570 nm: the maximum formazan absorbance wavelength; 630 nm: the background noise wavelength). The measured absorbance directly correlates to the number of viable cells. The cell viability was calculated as follows: cell viability (% RC) = (absorbance of treated cells/absorbance of control) × 100. The results of at least two independent experiments were expressed as the average of the percentage of the remaining viable cells (% RC) ± the standard error (SD).

### 4.5. Antibacterial Assays

#### 4.5.1. Essential Oil’s Direct Activity

The broth microdilution method in 96-well plates was used to obtain the minimum inhibitory concentration (MIC) of each essential oil according to Clinical and Laboratory Standards Institute (CLSI) standards [46]. The essential oils and different antibiotics were tested alone against all the strains mentioned above. Stock solutions were made in DMSO (5%) and diluted in the corresponding broth medium to achieve a final concentration of 2 mg/mL. Serial two-fold dilutions were made in order to obtain a range concentration of 1000 to 1.95 μg/mL for the essential oils and 64 to 1 μg/mL for the antibiotics. A suspension of 24-hour colonies was stirred in a physiological solution (0.85% NaCl) to obtain a turbidity equivalent to 0.5 McFarland and added to the wells to achieve a final concentration of 5 × 10^5^ CFU/mL. Growth and a non-growth controls were carried out. The plates were incubated at 37 °C overnight. Then, 30 μL (0.8 mg/mL) of MTT were used to determine by naked eye the minimum inhibitory concentration. All the tests were made in triplicate at least.

#### 4.5.2. Synergistic Activity between the Essential Oils and Antibiotics

The synergistic activity was determined for the association of an antibiotic with an essential oil both at an active concentration. The broth microdilution method was used to evaluate the fractional inhibitory concentration (FIC) and the FIC index (FICI) as described before [47]. The essential oils (EO) were combined with antibiotics (AB) in a 1:1 ratio and serially dilute (from 1000 μg/mL and 64 μg/mL, respectively) in the 96-well plates. The FICI was determined using the following equations: FICI = FIC _AB_ + FIC _EO_ where FIC _AB_ = MIC _AB in combination with the EO/_MIC _AB alone_ and FIC _EO_ = MIC _EO in combination with the AB/_MIC _EO alone_. The four possible types of interaction are: synergistic—S (FICI ≤ 0.5), additive—A (0.5 < FICI ≤1), indifferent—I (1 < FICI ≤ 2) and antagonist—An (FIC > 2) [48]. The tested strains (and the respective antibiotics tested) were *S. aureus* LMG 15975 (amoxicillin and penicillin V), *S. aureus* LMG 16217 (amoxicillin and penicillin V), *E. coli* LMG 15862 (amoxicillin and penicillin V) and *P. aeruginosa* LMG 6395 (doxycycline and tetracycline). All the tests were made in triplicate.

#### 4.5.3. Essential Oil’s Indirect Activity

The ability of essential oils at a sub-MIC concentration (non-active concentration) to restore or increase the activity of the antibiotics against resistant strains was determined by broth microdilution method [49]. The antibiotics were diluted by two-fold dilutions (64 to 0.007 μg/mL) and then the essential oils at sub-MIC were added to the wells. The essential oils are considered to have an indirect activity when the MIC of the association is lower than the MIC of the antibiotic alone. The tested strains (and the antibiotics) were *S. aureus* LMG 15975 (amoxicillin and penicillin V), *S. aureus* LMG 16217 (amoxicillin and penicillin V), *E. faecalis* LMG 8222 (cefotaxime, fosfomycin and gentamicin), *E. coli* LMG 15862 (amoxicillin and penicillin V), *P. aeruginosa* LMG 6395 (cefotaxime, doxycycline and tetracycline), *E. aerogenes* LMG 2094 (amoxicillin, cefotaxime and penicillin V) and *K. pneumoniae* LMG 20218 (ampicillin, cefotaxime and penicillin V). All the tests were made in triplicate.

#### 4.5.4. Direct and Indirect Activities of Each Major Compound of the Essential Oils

Using the same methods described in essential oil’s direct and indirect activities, the direct and indirect activities of the major compound of each essential oil were investigated. The chosen strain for this part of the research was *S. aureus* LMG 15975 and the antibiotic associated in the indirect assay was the penicillin V. All the tests were made in triplicate.

### 4.6. Statistical Analysis

The experiments were performed at least in duplicate, for both GC–MS analysis and cytotoxic activity, and at least in triplicate for the antibacterial assays. For each antibacterial assay, median values from all the independent measurements were calculated. For the percentages of GC–MS analyses and cytotoxic activity, data were expressed as mean values ± standard deviation (SD).

## 5. Conclusions

Regarding the three assays, all of the ten essential oils showed at some point an activity whether direct, indirect or/and synergistic. The activity of the Chinese cinnamon was very promising but its use in a topical or oral form is compromised regarding its toxicity on both the keratinocyte cell line—HaCaT and the epithelial cell line—CoN. Used at a sub-MIC concentration, the Spanish lavender and the palmarosa can be highlighted since they can restore the activity of the penicillin V against MRSA at a concentration close to the one active against an MSSA. Even when the sub-MIC is fixed at a non-toxic concentration, the German chamomile, the clove, the Spanish lavender, the oregano and the palmarosa can increase the activity of the penicillin V against the MRSA LMG 15975. These results prove once again that some essential oils are a very good source of antibacterial compounds and that they should be better investigated. One problem in the developing of essential oils in clinics is the high variability of their chemical composition, which depends on many factors, both biotic and abiotic factors. One big breakthrough would be to find a way to standardize the chemical composition of essential oils in order to have a stable mixture. Special attention should also be paid to the methods and the choice of strains. A universal guideline should be implemented with standardized evaluation criteria such as, for example, to always test a reference strain and perform controls.

## Figures and Tables

**Table 1:** Cytotoxicity of the ten essential oils on the keratinocyte cell line, HaCaT (% RC ± SD, *n* = 3) and on the epithelial cell line, CoN (% RC ± SD, *n* = 2). In bold, the non-cytotoxic results.

	% RC ± SD							
EO	HaCaT				CoN			
	16 μg/mL	32 μg/mL	125 μg/mL	250 μg/mL	16 μg/mL	32 μg/mL	125 μg/mL	250 μg/mL
AW	**129.71 ± 1.43**	**123.53 ± 4.86**	**103.14 ± 3.43**	2.73 ± 0.64	**91.72 ± 0.35**	**92.26 ± 1.94**	5.84 ± 0.55	2.18 ± 1.29
BE	**122.64 ± 1.58**	**111.40 ± 5.95**	**103.12 ± 4.15**	**98.87 ± 0.81**	**104.48 ± 2.58**	**94.80 ± 3.91**	**95.97 ± 1.19**	57.26 ± 0.13
CA	**103.13 ± 2.55**	**101.05 ± 0.71**	34.25 ± 2,00	2.90 ± 0.46	**101.76 ± 2.96**	**103.09 ± 2.78**	**102.66 ± 3.84**	7.92 ± 2.29
CC	7.58 ± 0.81	6.32 ± 1.42	4.12 ± 0.84	3.26 ± 0.83	28.36 ± 10.17	8.69 ± 0.50	3.16 ± 0.72	1.89 ± 0.19
CF	**102.22 ± 0.80**	**101.13 ± 0.56**	15.70 ± 1.32	2.98 ± 0.37	**98.22 ± 3.87**	19.52 ± 0.68	9.75 ± 0.09	3.75 ± 0.44
GF	**130.53 ± 0.22**	**116.44 ± 1.41**	71.87 ± 2.04	37.28 ± 1.08	**102.44 ± 5.57**	**99.89 ± 1.53**	**89.23 ± 4.66**	76.05 ± 4.55
LG	40.63 ± 0.38	21.54 ± 1.13	2.16 ± 0.75	1.77 ± 0.29	**95.30 ± 5.51**	**93.99 ± 3.24**	7.72 ± 2.44	3.41 ± 0.53
LS	**104.05 ± 7.91**	**98.82 ± 12.92**	76.25 ± 6.58	5.77 ± 2.23	**98.77 ± 14.52**	**85.18 ± 11.20**	62.09 ± 2.19	3.52 ± 0.93
OC	**85.95 ± 5.83**	**82.16 ± 4.93**	4.28 ± 1.51	3.74 ± 0.20	**97.43 ± 10.49**	**82.34 ± 18.55**	6.26 ± 0.42	5.44 ± 4.06
PM	**101.99 ± 0.47**	**98.68 ± 1.14**	**99.30 ± 1.30**	68.3 ± 4.36	**102.12 ± 0.20**	**99.81 ± 0.35**	**96.36 ± 4.39**	**93.63 ± 3.75**

AW—ajowan, BE—basil, CA—German chamomile, CC—Chinese cinnamon, CF—coriander, EO—essential oil, GF—clove, LG—lemongrass, LS—Spanish lavender, OC—oregano, PM—palmarosa, % RC—percentage of remaining living cells, SD—standard error.

**Table 2:** Direct activity of the essential oils against the Gram-negative strains (*n* = 6). In bold, the most promising results.

	Minimum Inhibitory Concentration (MIC, μg/mL)				
EO	*E. coli* LMG 8223	*E. coli* LMG 15862	*P. aeruginosa* LMG 6395	*K. pneumoniae* LMG 20218	*E. aerogenes* LMG 2094
**AW**	**500**	1000	>1000	**500**	**500**
BE	>1000	>1000	>1000	>1000	>1000
CA	>1000	>1000	>1000	>1000	>1000
**CC**	**250**	**500**	**500**	**250**	**250**
CF	1000	>1000	>1000	>1000	>1000
GF	1000	1000	>1000	1000	1000
LG	1000	>1000	>1000	>1000	>1000
LS	>1000	>1000	>1000	>1000	>1000
**OC**	**250**	**500**	**500**	**500**	**500**
PM	1000	>1000	>1000	>1000	1000
AMP	64	>64	>64	>64	>64
AMX	8	>64	>64	>64	>64
CTX	<1	<1	8	32	4
DOX	<1	<1	2	8	1
PEN V	>64	>64	>64	>64	>64
TET	<1	<1	2	8	2

AW—ajowan, BE—basil, CA—German chamomile, CC—Chinese cinnamon, CF—coriander, EO—essential oil, GF—clove, LG—lemongrass, LS—Spanish lavender, OC—oregano, PM—palmarosa, AMP—ampicillin, AMX—amoxicillin, CTX—cefotaxime, DOX—doxycycline, PEN V—penicillin V, TET—tetracycline.

**Table 3:** Direct activity of the essential oils against the Gram-positive strains (*n* = 6). In bold, the most promising results.

	Minimum Inhibitory Concentration (MIC, μg/mL)			
EO	*S. aureus* LMG 8064 (MSSA)	*S. aureus* LMG 15975 (MRSA)	*S. aureus* LMG 16217 (MRSA)	*E. faecalis* LMG 8222
**AW**	**500**	**500**	**500**	>1000
BE	>1000	>1000	>1000	>1000
CA	>1000	>1000	>1000	>1000
**CC**	**250**	**250**	**250**	**500**
**CF**	**125**	**125**	**125**	1000
GF	500	1000	1000	>1000
**LG**	**250**	**500**	**250**	>1000
LS	>1000	>1000	>1000	>1000
**OC**	**250**	**250**	**250**	1000
**PM**	**500**	1000	1000	>1000
AMX	0.03	8	64	< 1
CTX	< 1	2	>64	>64
FOF	4	16	16	64
GEN	< 1	>64	8	64
PEN V	0.015	4	64	1

AW—ajowan, BE—basil, CA—German chamomile, CC—Chinese cinnamon, CF—coriander, EO—essential oil, GF—clove, LG—lemongrass, LS—Spanish lavender, OC—oregano, PM—palmarosa, AMX—amoxicillin, CTX—cefotaxime, FOF—fosfomycin, GEN—gentamicin, PEN V—penicillin V.

**Table 4:** Synergistic activity of the essential oils combined with antibiotics, both at active concentrations in a 1:1 ratio against two MRSA, *E. coli* and *P. aeruginosa* (*n* = 3). In bold, the most promising results.

Combination between Essential Oils and Antibiotics																
	*S. aureus* LMG 15975				*S. aureus* LMG 16217				*E. coli* LMG 15862				*P. aeruginosa* LMG 6395			
EO	AMX		PEN V		AMX		PEN V		AMX		PEN V		DOX		TET	
	FICI	Int.	FICI	Int.	FICI	Int.	FICI	Int.	FICI	Int.	FICI	Int.	FICI	Int.	FICI	Int.
AW	0.75	A	0.63	A	0.75	A	0.75	A	1.50	I	1.50	I	1.13	I	1.13	I
BE	0.56	A	1.06	I	1.50	I	1.50	I	2.00	I	2.00	I	0.56	A	1.13	I
**CA**	0.56	A	0.53	A	0.75	A	**0.38**	**S**	2.00	I	2.00	I	0.56	A	1.13	I
**CC**	**0.50**	**S**	0.75	A	0.63	A	0.63	A	1.25	I	1.25	I	1.50	I	1.50	I
**CF**	**0.38**	**S**	1.00	A	0.56	A	1.13	I	2.00	I	2.00	I	0.56	A	1.13	I
**GF**	0.63	A	0.56	A	1.00	A	**0.50**	**S**	1.50	I	1.50	I	1.13	I	1.13	I
**LG**	0.63	A	0.56	A	**0.38**	**S**	0.75	A	2.00	I	2.00	I	0.56	A	1.13	I
LS	0.56	A	0.53	A	0.75	A	0.75	A	2.00	I	2.00	I	0.56	A	1.13	I
**OC**	**0.38**	**S**	0.63	A	0.63	A	1.25	I	1.25	I	1.25	I	1.50	I	1.50	I
PM	0.56	A	1.06	I	0.75	A	0.75	A	1.50	I	1.50	I	0.56	A	1.13	I

AW—ajowan, BE—basil, CA—German chamomile, CC—Chinese cinnamon, CF—coriander, EO—essential oil, GF—clove, LG—lemongrass, LS—Spanish lavender, OC—oregano, PM—palmarosa, AMX—amoxicillin, DOX—doxycycline, PEN V—penicillin V, TET—tetracycline, A—additive, FICI—Fractional Inhibitory Concentration Index, I—indifferent, Int.—interaction type, S—synergistic.

**Table 5:** Indirect activity of the essential oils combined at a non-active concentration (sub-MIC) with antibiotics, against *E. aerogenes* and *K. pneumoniae* (*n* = 3). In bold, the most promising results.

	Minimum Inhibitory Concentration (MIC, μg/mL)			
EO	*E. aerogenes* *LMG 2094*		*K. pneumoniae* *LMG 20218*	
	*EO* *Sub-MIC +*	CTX	*EO* *Sub-MIC +*	CTX
AB_alone_		4		32
AW	*125 +*	4	*125 +*	32
BE	*500 +*	4	*500 +*	32
CA	*500 +*	2	*500 +*	16
CC	*62.5 +*	2	*62.5 +*	32
CF	*500 +*	2	*500 +*	32
GF	*250 +*	2	*250 +*	16
LG	*500 +*	2	*500 +*	32
LS	*500 +*	4	*500 +*	**8**
OC	*125 +*	**1**	*125 +*	16
PM	*250 +*	4	*500 +*	32

AW—ajowan, BE—basil, CA—German chamomile, CC—Chinese cinnamon, CF—coriander, EO—essential oil, GF—clove, LG—lemongrass, LS—Spanish lavender, OC—oregano, PM—palmarosa, AB—antibiotic, CTX—cefotaxime, EO—essential oil.

**Table 6:** Indirect activity of the essential oils, combined at a non-active concentration (sub-MIC) with antibiotics, against the two MRSA and *E. faecalis* (*n* = 3). In bold, the most promising results.

	Minimum Inhibitory Concentration (MIC, μg/mL)										
EO	*S. aureus* LMG 15975					*S. aureus* LMG 16217			*E. faecalis* LMG 8222		
	*EO* *Sub-MIC +*	AMX	PEN V	*EO* *Sub-MIC +*	PEN V	*EO* *Sub-MIC +*	AMX	PEN V	*EO* *Sub-MIC +*	FOF	GEN
AB_alone_		8	4		4		64	64		64	64
AW	125 +	**1**	**0.5**	*32 +*	2	*125 +*	32	64	*500 +*	64	**1**
BE	500 +	**2**	2	*32 +*	4	*500 +*	64	64	*500 +*	64	64
CA	500 +	**2**	**0.125**	*32 +*	**1**	*500 +*	32	32	*500 +*	64	**1**
CC	62.5 +	**2**	**1**	*nt*	nt	*62.5 +*	64	64	*125 +*	64	**8**
CF	31.3 +	**2**	2	*nt*	nt	*31.3 +*	64	64	*250 +*	64	**2**
GF	250 +	**1**	**0.125**	*32 +*	**1**	*250 +*	32	64	*500 +*	64	**4**
LG	125 +	**2**	**0.25**	*nt*	nt	*62.5 +*	64	32	*500 +*	64	**1**
LS	500 +	**1**	**0.06**	*32 +*	**1**	*500 +*	32	32	*500 +*	32	**8**
OC	62.5 +	**2**	**0.25**	*32 +*	**0.5**	*62.5 +*	64	64	*250 +*	64	**2**
PM	250 +	**2**	**0.06**	*32 +*	**1**	*250 +*	64	64	*500 +*	64	**4**

AW—ajowan, BE—basil, CA—German chamomile, CC—Chinese cinnamon, CF—coriander, EO—essential oil, GF—clove, LG—lemongrass, LS—Spanish lavender, OC—oregano, PM—palmarosa, AB—antibiotic, AMX—amoxicillin, EO—essential oil, FOF—Fosfomycin, GEN—gentamicin, PEN V—penicillin V, nt—not tested.

**Table 7:** Geographical origin, organ and chemical composition (compounds above 10%) (*n* = 2) of the ten tested essential oils. In bold, the main compound.

EO	Origin of the Plant	Organ Used	Chemical Composition	
			Compound of the EO	% in the EO ± SD
AW	India	fruit	**thymol**	**45.17 ± 1.94**
			γ-terpinene	24.16 ± 0.01
			p-cymene	23.16 ± 1.69
BE	India	flowered top	**estragole**	**87.58 ± 2.22**
CA	U.K.	flower	**E-β-farnesene**	**34.61 ± 3.79**
CC	China	branch	**E-cinnamaldehyde**	**82.05 ± 10.46**
CF	U.K.	leaf	**linalool**	**43.67 ± 4.16**
			2-decenal	11.07 ± 0.53
GF	Indonesia	flower bud	**eugenol**	**84.58 ± 0.35**
LG	Guatemala	aerial parts	**citral**	**75.16 ± 6.60**
LS	France	flowered top	**camphor**	**32.54 ± 2.75**
			fenchone	24.40 ± 0.33
OC	Morocco	flowered top	**carvacrol**	**57.21 ± 2.04**
			thymol	13.73 ± 3.71
			p-cymene	11.55 ± 0.74
PM	Nepal	aerial parts	**geraniol**	**81.05 ± 0.70**
			geranyl acetate	11.01 ± 0.30

AW—ajowan, BE—basil, CA—German chamomile, CC—Chinese cinnamon, CF—coriander, GF—clove, LG—lemongrass, LS—Spanish lavender, OC—oregano, PM—palmarosa, EO—essential oil, SD—standard deviation.

**Table 8:** Direct and indirect activity of the major compounds of the essential oils tested (*n* = 3). In bold, the most promising results.

			Minimum Inhibitory Activity (MIC, μg/mL)					
			*S. aureus* LMG 15975					
EO			Direct Activity		Indirect Activity			
		% of the Compound in the EO	EO Compound	EO	EO Compound		EO	
					*Sub-MIC +*	PEN V *	*Sub-MIC +*	PEN V *
AW	Thymol	45.17	**250**	**500**	**62..5 +**	**0.125**	**125 +**	**0.5**
BE	Estragole	87.58	>1000	>1000	500 +	2	500 +	2
CA	E-β-farnesene	34.61	>1000	>1000	**500 +**	**0.06**	**500 +**	**0.125**
CC	E-cinnamaldehyde	82.05	**250**	**250**	62.5 +	4	62.5 +	1
CF	2-decenal	11.07	**250**	**125**	**62.5 +**	**0.06**	31.3 +	2
GF	Eugenol	84.58	1000	1000	250 +	1	**250 +**	**0.125**
LG	Citral	75.16	**500**	**500**	**125 +**	**0.06**	**125 +**	**0.25**
LS	Camphor	32.54	>1000	>1000	500 +	1	**500 +**	**0.06**
OC	Carvacrol	57.21	**125**	**250**	**31.3 +**	**0.5**	**62.5 +**	**0.25**
PM	Geraniol	81.05	1000	1000	**250 +**	**0.03**	**250 +**	**0.06**

* MIC PEN V alone = 4 μg/mL. AW—ajowan, BE—basil, CA—German chamomile, CC—Chinese cinnamon, CF—coriander, GF—clove, LG—lemongrass, LS—Spanish lavender, OC—oregano, PM—palmarosa, EO—essential oil, PEN V—penicillin V.

## Supplementary Materials

The following are available online at https://www.mdpi.com/2079-6382/9/10/717/s1, Table S1a–d: details values and the FIC calculations obtained for the synergistic activity. Figure S1: GC-MS analysis of the ajowan provided by the manufacturer; Figure S2: GC-MS analysis of the basil provided by the manufacturer; Figure S3: GC-MS analysis of the German chamomile provided by the manufacturer; Figure S4: GC-MS analysis of the Chinese cinnamon provided by the manufacturer; Figure S5: GC-MS analysis of the coriander provided by the manufacturer; Figure S6: GC-MS analysis of the clove provided by the manufacturer; Figure S7: GC-MS analysis of the lemongrass provided by the manufacturer; Figure S8: GC-MS analysis of the Spanish lavender provided by the manufacturer; Figure S9: GC-MS analysis of the oregano provided by the manufacturer; Figure S10: GC-MS analysis of the palmarosa provided by the manufacturer.
