# Supplementary material for: Antibacterial and Cytotoxic Activities of Ten Commercially Available Essential Oils
Source: Antibiotics (Basel). 2020 Oct 20;9(10):717. doi: 10.3390/antibiotics9100717 (PMC7589993; doi:10.3390/antibiotics9100717)
Supplement: Supplementary file 1 [file antibiotics-09-00717-s001.pdf]

Supplementary Material

Antibacterial and cytotoxic activities of ten essential oils commercially available

Sofia Oliveira Ribeiro [1], Véronique Fontaine [2], Véronique Mathieu [3], Abdesselam Zhiri [4,5], Dominique Baudoux [4], Caroline Stévigny [*,1] and Florence Souard [*,6,7]

[1] Department of Research in Drug Development (RD3), Pharmacognosy, Bioanalysis and Drug Discovery Unit, Faculty of Pharmacy, Université libre de Bruxelles, Boulevard du Triomphe, 1050 Brussels, Belgium
[2] Department of Research in Drug Development (RD3), Microbiology, Bioorganic and Macromolecular Chemistry Unit, Faculty of Pharmacy, Université libre de Bruxelles (ULB), Boulevard du Triomphe, 1050 Brussels, Belgium
[3] Department of Pharmacotherapy and Pharmaceutics, Université libre de Bruxelles (ULB), Boulevard du Triomphe, 1050 Brussels, Belgium
[4] Pranarôm International S.A. 37, Avenue des Artisans, 7822, Ghislenghien, Belgium
[5] Unité de recherche en Biotechnologie végétale, Université libre de Bruxelles, CP 300, Rue Prof. Jeener & Brachet 12, 6041 Gosselies, Belgium
[6] Department of Pharmacotherapy and Pharmaceutics (DPP), Pharmacology, Pharmacotherapy and Pharmaceutical care Unit, Faculty of Pharmacy, Université libre de Bruxelles (ULB), Boulevard du Triomphe, 1050 Brussels, Belgium
[7] Université Grenoble Alpes, DPM UMR 5063, F3Y041, Grenoble, France.
* Equally contributing project leaders

Corresponding author: Sofia Oliveira Ribeiro
Tel.: +32 02 650 52 98
E-mail address: sofia.marilia.oliveira.ribeiro@ulb.be

Materials and methods

Synergistic activity between the essential oils and antibiotics

As mentioned in the article, we tested the synergistic activity between the essential oils and common antibiotics by evaluating the Fractional Inhibitory Concentration (FIC) and the FIC index (FICI). The essential oils (EO) were combined with antibiotics (AB) in a 1:1 ratio and serially dilute (from 1000 $\mu g.ml^{-1}$ and 64 $\mu g.ml^{-1}$, respectively) in the 96-well plates. The FICI was determined using the following equations:

$$FICI = FIC_{AB} + FIC_{EO}$$

$$\text{where : } FIC_{AB} = MIC_{AB\text{ in combination with the EO}} / MIC_{AB\text{ alone}}$$

$$FIC_{EO} = MIC_{EO\text{ in combination with the AB}} / MIC_{EO\text{ alone}}$$

The four possible types of interaction are :

1. synergy (FICI ≤ 0,5)
2. additive (0,5 < FICI ≤1)
3. indifferent (1 < FICI ≤ 2)

4. antagonist (FIC > 2)

The tested strains (and the respective antibiotics tested) were *S. aureus* LMG 15975 (amoxicillin and penicillin V), *S. aureus* LMG 16217 (amoxicillin and penicillin V), *E. coli* LMG 15862 (amoxicillin and penicillin V) and *P. aeruginosa* LMG 6395 (doxycycline and tetracycline). The direct activity leading to obtain the MIC_{AB} and the MIC_{EO} were made at the same time as the synergistic test where we obtained the $MIC_{AB/EO}$ and the $MIC_{EO/AB}$. The tests were made in triplicate.

Figure of contents

Table S1a – Synergistic activity of the ten commercial essential oils (EO) in association with two different antibiotics (AB), the amoxicillin (AMX) and the penicillin V (PEN V) against *Staphylococcus aureus* LMG 15975 (n=3).

Table S1b – Synergistic activity of the ten commercial essential oils (EO) in association with two different antibiotics (AB), the amoxicillin (AMX) and the penicillin V (PEN V) against *Staphylococcus aureus* LMG 16217 (n=3).

Table S1c – Synergistic activity of the ten commercial essential oils (EO) in association with two different antibiotics (AB), the amoxicillin (AMX) and the penicillin V (PEN V) against *Escherichia coli* LMG 15862 (n=3).

Table S1d – Synergistic activity of the ten commercial essential oils (EO) in association with two different antibiotics (AB), the doxycycline (DOX) and the tetracycline (TET) against *Pseudomonas aeruginosa* LMG 6395 (n=3).

Figure S1 – Certificate of the chemical analysis for the lot number OF17650 of *Trachyspermum ammi* L. (ajowan) provided by Pranarôm. Screen capture made on 1st September 2020.

Figure S2 – Certificate of the chemical analysis for the lot number OF22779 of *Ocimum basilicum ssp basilicum* L. (basil) provided by Pranarôm. Screen capture made on 1st September 2020.

Figure S3 – Certificate of the chemical analysis for the lot number OF22830 of *Matricaria recutita* L. (German chamomile) provided by Pranarôm. Screen capture made on 1st September 2020.

Figure S4 – Certificate of the chemical analysis for the lot number OF22426 of *Cinnamomum cassia* (L.) J. Presl. (Chinese cinnamon) provided by Pranarôm. Screen capture made on 1st September 2020.

Figure S5 – Certificate of the chemical analysis for the lot number OF21374 of *Coriandrum sativum* L. (coriander) provided by Pranarôm. Screen capture made on 1st September 2020.

Figure S6 – Certificate of the chemical analysis for the lot number OF22293 of *Eugenia caryophyllus* (Spreng.) Bullock & S. G. Harrison. (clove) provided by Pranarôm. Screen capture made on 1st September 2020.

Figure S7 – Certificate of the chemical analysis for the lot number OF20407 of *Cymbopogon citratus (DC.) Staph.* (lemongrass) provided by Pranarôm. Screen capture made on 1st September 2020.

Figure S8 – Certificate of the chemical analysis for the lot number OF19205 of *Lavandula stoechas* L. (Spanish lavender) provided by Pranarôm. Screen capture made on 1st September 2020.

Figure S9 – Certificate of the chemical analysis for the lot number OF22429 of *Origanum compactum* Benth. (oregano) provided by Pranarôm. Screen capture made on 1st September 2020.

Figure S10 – Certificate of the chemical analysis for the lot number OF21237 of *Cymbopogon martinii var. motia* (Roxb.) W.Watson (palmarosa) provided by Pranarôm. Screen capture made on 1st September 2020.

Results

Table S1a – Synergistic activity of the ten commercial essential oils (EO) in association with two different antibiotics (AB), the amoxicillin (AMX) and the penicillin V (PEN V) against *Staphylococcus aureus* LMG 15975 (n=3).

S. aureus LMG 15975									
AB	EO	MIC $_{AB}$ ($\mu g.ml^{-1}$)	MIC $_{EO}$ ($\mu g.ml^{-1}$)	MIC $_{AB/EO}$ ($\mu g.ml^{-1}$)	MIC $_{EO/AB}$ ($\mu g.ml^{-1}$)	FIC $_{AB}$	FIC $_{EO}$	FIC	Interaction
AMX	/AW	16	500	8	125	0,50	0,25	0,75	Additive
	/BE	16	2000	8	125	0,50	0,06	0,56	Additive
	/CA	16	2000	8	125	0,50	0,06	0,56	Additive
	/CC	16	250	4	62,5	0,25	0,25	0,50	Synergy
	/CF	16	125	2	31,25	0,13	0,25	0,38	Synergy
	/GF	16	1000	8	125	0,50	0,13	0,63	Additive
	/LG	16	1000	8	125	0,50	0,13	0,63	Additive
	/LS	16	2000	8	125	0,50	0,06	0,56	Additive
	/OC	16	500	4	62,5	0,25	0,13	0,38	Synergy
	/PM	16	2000	8	125	0,50	0,06	0,56	Additive
PEN V	/AW	8	500	4	62,5	0,50	0,13	0,63	Additive
	/BE	8	2000	8	125	1,00	0,06	1,06	Indifferent
	/CA	8	2000	4	62,5	0,50	0,03	0,53	Additive
	/CC	8	250	4	62,5	0,50	0,25	0,75	Additive
	/CF	8	125	4	62,5	0,50	0,50	1,00	Additive
	/GF	8	1000	4	62,5	0,50	0,06	0,56	Additive
	/LG	8	1000	4	62,5	0,50	0,06	0,56	Additive
	/LS	8	2000	4	62,5	0,50	0,03	0,53	Additive
	/OC	8	500	4	62,5	0,50	0,13	0,63	Additive
	/PM	8	2000	8	125	1,00	0,06	1,06	Indifferent

Table S2b – Synergistic activity of the ten commercial essential oils (EO) in association with two different antibiotics (AB), the amoxicillin (AMX) and the penicillin V (PEN V) against *Staphylococcus aureus* LMG 16217 (n=3).

S. aureus LMG 16217									
AB	EO	MIC $_{AB}$ ($\mu g.ml^{-1}$)	MIC $_{EO}$ ($\mu g.ml^{-1}$)	MIC $_{AB/EO}$ ($\mu g.ml^{-1}$)	MIC $_{EO/AB}$ ($\mu g.ml^{-1}$)	FIC $_{AB}$	FIC $_{EO}$	FIC	Interaction
AMX	/AW	64	500	16	250	0,25	0,50	0,75	Additive
	/BE	64	2000	64	1000	1,00	0,50	1,50	Indifferent
	/CA	64	2000	32	500	0,50	0,25	0,75	Additive
	/CC	64	250	8	125	0,13	0,50	0,63	Additive
	/CF	64	125	4	62,5	0,06	0,50	0,56	Additive
	/GF	64	1000	32	500	0,50	0,50	1,00	Indifferent
	/LG	64	500	8	125	0,13	0,25	0,38	Synergy
	/LS	64	2000	32	500	0,50	0,25	0,75	Additive
	/OC	64	250	8	125	0,13	0,50	0,63	Additive
	/PM	64	2000	32	500	0,50	0,25	0,75	Additive
PEN V	/AW	64	500	16	250	0,25	0,50	0,75	Additive
	/BE	64	2000	64	1000	1,00	0,50	1,50	Indifferent
	/CA	64	2000	16	250	0,25	0,13	0,38	Synergy

	/CC	64	250	8	125	0,13	0,50	0,63	Additive
	/CF	64	125	8	125	0,13	1,00	1,13	Indifferent
	/GF	64	1000	16	250	0,25	0,25	0,50	Synergy
	/LG	64	500	16	250	0,25	0,50	0,75	Additive
	/LS	64	2000	32	500	0,50	0,25	0,75	Additive
	/OC	64	250	16	250	0,25	1,00	1,25	Indifferent
	/PM	64	2000	32	500	0,50	0,25	0,75	Additive

Table S3c – Synergistic activity of the ten commercial essential oils (EO) in association with two different antibiotics (AB), the amoxicillin (AMX) and the penicillin V (PEN V) against *Escherichia coli* LMG 15862 (n=3).

E. coli LMG 15862									
AB	EO	MIC AB (µg.ml-1)	MIC EO (µg.ml-1)	MIC AB/EO (µg.ml-1)	MIC EO/AB (µg.ml-1)	FIC AB	FIC EO	FIC	Interaction
AMX	/AW	128	1000	64	1000	0,50	1,00	1,50	Indifferent
	/BE	128	2000	128	2000	1,00	1,00	2,00	Indifferent
	/CA	128	2000	128	2000	1,00	1,00	2,00	Indifferent
	/CC	128	500	32	500	0,25	1,00	1,25	Indifferent
	/CF	128	2000	128	2000	1,00	1,00	2,00	Indifferent
	/GF	128	1000	64	1000	0,50	1,00	1,50	Indifferent
	/LG	128	2000	128	2000	1,00	1,00	2,00	Indifferent
	/LS	128	2000	128	2000	1,00	1,00	2,00	Indifferent
	/OC	128	500	32	500	0,25	1,00	1,25	Indifferent
	/PM	128	1000	64	1000	0,50	1,00	1,50	Indifferent
PEN V	/AW	128	1000	64	1000	0,50	1,00	1,50	Indifferent
	/BE	128	2000	128	2000	1,00	1,00	2,00	Indifferent
	/CA	128	2000	128	2000	1,00	1,00	2,00	Indifferent
	/CC	128	500	32	500	0,25	1,00	1,25	Indifferent
	/CF	128	2000	128	2000	1,00	1,00	2,00	Indifferent
	/GF	128	1000	64	1000	0,50	1,00	1,50	Indifferent
	/LG	128	2000	128	2000	1,00	1,00	2,00	Indifferent
	/LS	128	2000	128	2000	1,00	1,00	2,00	Indifferent
	/OC	128	500	32	500	0,25	1,00	1,25	Indifferent
	/PM	128	1000	64	1000	0,50	1,00	1,50	Indifferent

Table S4d – Synergistic activity of the ten commercial essential oils (EO) in association with two different antibiotics (AB), the doxycycline (DOX) and the tetracycline (TET) against *Pseudomonas aeruginosa* LMG 6395 (n=3).

P. aeruginosa LMG 6395									
AB	EO	MIC AB (µg.ml-1)	MIC EO (µg.ml-1)	MIC AB/EO (µg.ml-1)	MIC EO/AB (µg.ml-1)	FIC AB	FIC EO	FIC	Interaction
DOX	/AW	16	2000	16	250	1,00	0,13	1,13	Indifferent
	/BE	16	2000	8	125	0,50	0,06	0,56	Additive
	/CA	16	2000	8	125	0,50	0,06	0,56	Additive
	/CC	16	500	16	250	1,00	0,50	1,50	Indifferent
	/CF	16	2000	8	125	0,50	0,06	0,56	Additive
	/GF	16	2000	16	250	1,00	0,13	1,13	Indifferent
	/LG	16	2000	8	125	0,50	0,06	0,56	Additive
	/LS	16	2000	8	125	0,50	0,06	0,56	Additive

	/OC	16	500	16	250	1,00	0,50	1,50	Indifferent
	/PM	16	2000	8	125	0,50	0,06	0,56	Additive

TET	/AW	16	2000	16	250	1,00	0,13	1,13	Indifferent
	/BE	16	2000	16	250	1,00	0,13	1,13	Indifferent
	/CA	16	2000	16	250	1,00	0,13	1,13	Indifferent
	/CC	16	500	16	250	1,00	0,50	1,50	Indifferent
	/CF	16	2000	16	250	1,00	0,13	1,13	Indifferent
	/GF	16	2000	16	250	1,00	0,13	1,13	Indifferent
	/LG	16	2000	16	250	1,00	0,13	1,13	Indifferent
	/LS	16	2000	16	250	1,00	0,13	1,13	Indifferent
	/OC	16	500	16	250	1,00	0,50	1,50	Indifferent
	/PM	16	2000	16	250	1,00	0,13	1,13	Indifferent

Figure S1 (page 1/4) – Certificate of the chemical analysis for the lot number OF17650 of *Trachyspermum ammi* L. (ajowan) provided by Pranarôm. Screen capture made 1st September 2020.

PRANARÔM
international

FICHE D'ANALYSE – ANALYSIS SHEET

Huile essentielle – Essential oil

Nom botanique – botanical name : ***Trachyspermum ammi***
Nom commun – french name: AJOWAN
Numéro du lot - lot number: **OF17650**
Origine - origin: ---- PRANARÔM - INDE
Partie de la plante – part of the plant: FRUIT
Date de distillation – distillation date : 06/2014
Date de péremption – out of date : 09/2019

Caractéristiques d'analyse – analysis characteristics:

CPG - SM HEWLETT PACKARD / CPG-FID
Colonne : HP INNOWAX 60-0.5-0.25
Programmation de température : 6 mn à 50°C -2°C/mn→250°C-20mn à 250°C
Gaz vecteur He : 22 psis

Caractéristiques physiques – physical characteristics :

Aspect – physical state	Liquide limpide
Couleur - colour	Jaune d'or
Odeur - odour	Phénolique, caractéristique
Densité à 20°C - density	0,910
Densité à 15°C - density	0,914
Indice de réfraction à 20°C - refractive index	1,497 5
Pouvoir rotatoire à 20°C – optical rotation	- 1 °
Miscibilité à l'éthanol à 80% - miscibility	1,4 volumes d'alcool / 1 volume d'HE (légère opalescence)
Point d'éclair : SETAFLASH - flashpoint	58,0 °C

Analyses pesticides – pesticide analysis :

Pesticides Organochlorés : **Dosage par GC MS détecteur XSD (méthode multirésidus interne validée selon la norme NF V03-110)** **Liste des pesticides recherchés (Pharmacopée Européenne)**: Alachlor, Aldrine, Bromophos Ethyl, Bromophos Methyl, Chlordane, Chlorfenvinphos, Chlorpyriphos, Chlorpyriphos Methyl, Chlorthal Dimethyl, Cyfluthrine, Cyhalothrine lambda, Cypermethrine, Dichlofluanide, Dichlorvos, Dicofol (Kelthane), Dieldrine, Endosulfan, Endosulfan sulfate, Endrine, Fenchlorphos (Ronnel), Fenchlorphos-oxon, Fenvalerate, Fluvalinate, Heptachlor, Heptachlor epoxide, Hexachlorobenzene, Hexachlorocyclohexane α, Hexachlorocyclohexane β, Hexachlorocyclohexane δ, Hexachlorocyclohexane ε, Lindane, Methoxychlore, Mirex, Naled, o,p'-DDD , o,p'-DDE, o,p'-DDT, Oxychlordane, p,p'-DDD, p,p'-DDE, p,p'-DDT, Pentachloroaniline, Pentachloroanisole, Permethrine , Phosalone, Procymidone, Profenophos, Prothiofos, Quintozene, S421, Tecnazene, Tetradifon, Vinclozoline	<u>Résultats</u> < LMR* * Limite Maximale de Résidus autorisée
Pesticides Organophosphorés : **Dosage par GC MS détecteur FPD (méthode multirésidus interne validée selon la norme NF V03-110)** **Liste des pesticides recherchés (Pharmacopée Européenne)**: Acephate, Azinphos Ethyl, Azinphos Méthyl, Bromophos Ethyl, Bromophos Méthyl, Chlorfenvinphos, Chlorpyriphos, Chlorpyriphos Methyl, Diazinon, Dichlorvos, Dimethoate, Ethion, Etrimphos, Fenchlorphos (Ronnel), Fenchlorphos-oxon, Fenitrothion, Fensulfothion (Dasanit), Fensulfothion-oxon, Fensulfothion-oxon-sulfone, Fensulfothion-sulfone, Fenthion, Fenthion-oxon, Fenthion-oxon-sulfone, Fenthion-oxon-sulfoxyde, Fenthion-sulfone, Fenthion-sulfoxyde, Fonofos, Malaoxon, Malathion, Mecarbam, Methacrifos, Methamidophos (Monitor), Methidathion, Monocrotophos, Naled, Omethoate, Paraoxon, Paraoxon Méthyl, Parathion Ethyl, Parathion Methyl, Phosalone, Phosmet, Pirimiphos Ethyl, Pirimiphos Méthyl, Profenophos, Prothiofos, Quinalphos.	<u>Résultats</u> < LMR* * Limite Maximale de Résidus autorisée

Profil CHROMATOGRAPHIQUE

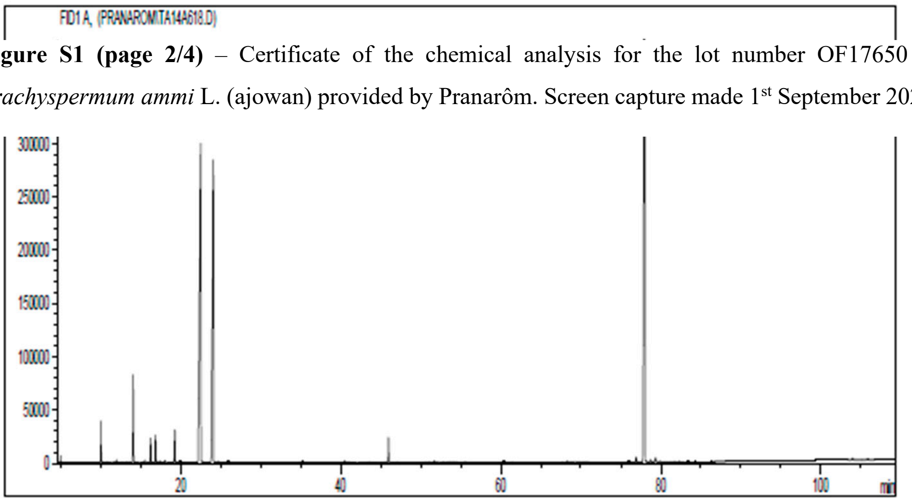

Figure S1 (page 2/4) – Certificate of the chemical analysis for the lot number OF17650 of *Trachyspermum ammi* L. (ajowan) provided by Pranarôm. Screen capture made 1st September 2020.

Tableau de résultats 1 : AJOWAN INDE

LOT N° OF17650

Pics	Temps de rétention	Constituants	%
1	5,0	ETHANOL	0,09
2	9,8	p-MENTHANE	0,01
3	10,0	α-PINENE	1,18
4	10,8	MENTHANE ISOMERE	0,02
5	11,6	α-FENCHENE	0,02
6	12,0	CAMPHENE	0,07
7	13,2	MENTHENE ISOMERE	0,01
8	14,1	**β-PINENE**	**3,04**
9	14,7	PINADIENE	0,01
10	15,3	Δ2-CARENE	0,02
11	15,5	p-MENTH-2-ENE	0,09
12	16,3	Δ3-CARENE	0,79
13	16,9	β-MYRCENE	0,84
18	19,3	LIMONENE	1,15
19	19,9	1,8-CINEOLE	0,06
20	20,0	β-PHELLANDRENE	0,05
21	22,5	**γ-TERPINENE**	**27,44**
22	24,1	**p-CYMENE**	**20,55**
23	24,7	TERPINOLENE	0,05
24	25,9	CYMENE ISOMERE	0,03
25	35,0	CETONE TERPENIQUE	0,02
26	35,2	α,p-DIMETHYLSTYRENE	0,06
27	35,5	MENTHATRIENE ISOMERE	0,01
28	40,3	EPOXYDE TERPENIQUE	0,03
29	40,5	EPOXYDE TERPENIQUE	0,07
30	41,0	EPOXYDE TERPENIQUE ISOMERE	0,02
31	43,7	4-CARANONE	0,01
32	44,1	TERPINENE-1-OL	0,02
33	44,5	FENCHOL	0,03
34	45,6	HYDRATE DE CAMPHENE	0,02
35	46,0	TERPINENE-4-OL	0,92
36	48,4	BICYCLOHEPTYL BUTANONE Mw=166	0,01
37	49,2	Trans-PINOCARVEOL	0,02
38	50,0	ALCOOL TERPENIQUE	0,01
39	50,6	Trans-VERBENOL	0,01
40	51,0	NERAL	0,02
41	51,7	α-TERPINEOL	0,06
42	52,0	BORNEOL	0,02
43	52,8	ESTER TERPENIQUE	0,03
44	54,0	PIPERITONE	0,01
45	54,5	CARVONE	0,01

Figure S1 (page 3/4) – Certificate of the chemical analysis for the lot number OF17650 of *Trachyspermum ammi* L. (ajowan) provided by Pranarôm. Screen capture made 1st September 2020.

Tableau de résultats 2 : AJOWAN INDE

LOT N° OF17650

Pics	Temps de rétention	Constituants	%
46	57,3	MYRTENOL	0,01
47	59,7	Trans-CARVEOL	0,02
48	60,4	p-CYMENE-8-OL	0,04
49	65,6	PIPERITENONE	0,02
50	67,6	COMPOSÉ PHENOLIQUE	0,01
51	68,3	β-METHYL BENZENE PROPANOL	0,03
52	68,6	COMPOSÉ THYMOL ETHER	0,01
53	69,0	METHYL ETHYL PHENOL Mw=178	0,01
54	69,5	METHYL DIISOPROPYL PHENOL ISOMERE Mw=192	0,01
55	70,9	SESQUITERPENOL	0,02
56	71,7	p-MENTHA-1,4-DIEN-7-OL	0,02
57	73,1	p-CRESOL	0,01
58	74,1	CUMINOL	0,03
59	76,0	MENTHADIENOL ISOMERE	0,04
60	76,9	ISOTHYMOL	0,18
61	78,0	**THYMOL**	**41,92**
62	78,8	ISOCARVACROL	0,09
63	79,2	GERANYLGERANIADIENE ISOMERE	0,06
64	79,4	CARVACROL	0,18
65	79,9	COMPOSÉ PHENOLIQUE	0,02
66	81,8	METHYL PROPYL PHENOL ISOMERE	0,02
67	83,4	COMPOSÉ PHENOLIQUE	0,01
68	84,3	COMPOSÉ AROMATIQUE Mw=192	0,01
69	88,1	COMPOSÉ AROMATIQUE	0,05
70	88,3	COMPOSÉ PHENOLIQUE	0,02
71	90,6	COMPOSÉ AROMATIQUE	0,02
[illegible]	[illegible]	[illegible]	[illegible]

Figure S1 (page 4/4) – Certificate of the chemical analysis for the lot number OF17650 of *Trachyspermum ammi* L. (ajowan) provided by Pranarôm. Screen capture made 1st September 2020.

Pranarôm Int.
C. Schulze
Contrôle qualité

PRANARÔM
international

FICHE D'ANALYSE – ANALYSIS SHEET

Huile essentielle – Essential oil

Nom botanique – botanical name :	*Ocimum basilicum Organic*
Nom commun – french name :	BASILIC EXOTIQUE BIOLOGIQUE
Numéro du lot – lot number :	**OF22779**
Origine - origin : ---	PRANARÔM - INDE
Partie de la plante – part of the plant :	SOMMITÉ FLEURIE
Date de distillation – distillation date :	06/2015
Date de p éremption – out of date :	04/2021

Caractéristiques d'analyse – analysis characteristics:

CPG - SM HEWLETT PACKARD / CPG-FID
Colonne : HP INNOWAX 60-0.5-0.25
Programmation de température : 6 mn à 50°C –2 °C/mn→250°C -10mn à 250°C
Gaz vecteur He : 22 psis

Caractéristiques physiques – physical characteristics:

Aspect – physical state	Liquide limpide
Couleur - colour	Jaune très clair
Odeur - odour	Caractéristique, épicée
Densité à 20°C - density	0,938
Densité à 15°C - density	0,942
Indice de réfraction à 20°C – refractive index	1,504 7
Pouvoir rotatoire à 20°C - optical rotation	-7,5 °C
Miscibilité à l'éthanol à 80% - miscibility	2,5 volumes d'alcool à 80% / 1 volume d'HE
Point d'éclair : SETAFLASH - flashpoint	80,7 °C

Analyses pesticides – pesticide analysis :

Pesticides Organochlorés : Dosage par GC MS détecteur XSD (méthode multirésidus interne validée selon la norme NF V03-110)	Résultats
**Liste des pesticides recherchés (Pharmacopée Européenne):** Alachlor, Aldrine, Bromophos Ethyl, Bromophos Methyl, Chlordane, Chlorfenvinphos, Chlorpyriphos, Chlorpyriphos Methyl, Chlorthal Dimethyl, Cyfluthrine, Cyhalothrine lambda, Cypermethrine, Dichlofluanide, Dichlorvos, Dicofol (Kelthane), Dieldrine, Endosulfan, Endosulfan sulfate, Endrine, Fenchlorphos (Ronnel), Fenchlorphos-oxon, Fenvalerate, Fluvalinate, Heptachlor, Heptachlor epoxide, Hexachlorobenzene, Hexachlorocyclohexane α, Hexachlorocyclohexane β, Hexachlorocyclohexane δ, Hexachlorocyclohexane ε, Lindane, Methoxychlore, Mirex, Naled, o,p'-DDD , o,p'-DDE, o,p'-DDT, Oxychlordane, p,p'-DDD, p,p'-DDE, p,p'-DDT, Pentachloroaniline, Pentachloroanisole, Permethrine , Phosalone, Procymidone, Profenophos, Prothiofos, Quintozene, S421, Tecnazene, Tetradifon, Vinclozoline	< LMR*<br><br>* Limite Maximale de Résidus autorisée
**Pesticides Organophosphorés : Dosage par GC MS détecteur FPD (méthode multirésidus interne validée selon la norme NF V03-110)**	**Résultats**
[illegible] Méthyl, Parathion Ethyl, Parathion Methyl, Phosalone, Phosmet, Pirimiphos Ethyl, Pirimiphos Méthyl, Profenophos, Prothiofos, Quinalphos.	[illegible]<br><br>* Limite Maximale de Résidus autorisée

Figure S2 (page 1/4) – Certificate of the chemical analysis for the lot number OF22779 of *Ocimum basilicum ssp basilicum* L. (basil) provided by Pranarôm. Screen capture made on 1st September 2020.

Profil CHROMATOGRAPHIQUE

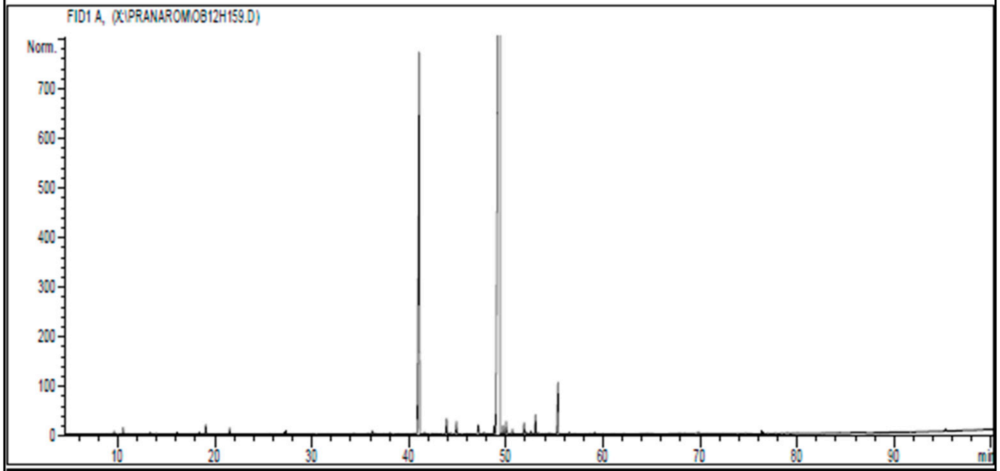

Figure S2 (page 2/4) – Certificate of the chemical analysis for the lot number OF22779 of *Ocimum basilicum ssp basilicum* L. (basil) provided by Pranarôm. Screen capture made on 1st September 2020.

Figure S2 (page 3/4) – Certificate of the chemical analysis for the lot number OF22779 of *Ocimum basilicum ssp basilicum* L. (basil) provided by Pranarôm. Screen capture made on 1st September 2020.

Tableau de résultats 1 : BASILIC BIO

LOT N° OF22779

Pics	Temps de rétention	Constituants	%
1	5,1	ACETONE	0,01
2	6,6	ISOVALERALDEHYDE	0,01
3	6,8	ETHANOL	0,01
4	7,4	3-METHYL-2-BUTENAL	0,01
5	9,6	α-PINENE	0,08
6	9,8	α-THUYENE	0,01
7	9,9	TOLUENE	0,01
8	10,4	COMPOSÉ Mw=138	0,01
9	10,6	DIMETHYL METHYLETHYL CYCLOPENTENE ISOMERE	0,17
10	10,7	COMPOSÉ TERPENIQUE	0,01
11	11,1	DIMETHYL METHYLETHYL CYCLOPENTENE ISOMERE	0,01
12	11,8	MENTHENE ISOMERE	0,01
13	12,9	2,5-DIMETHYL-1,6-HEPTADIENE	0,01
14	13,4	β-PINENE	0,06
15	14,0	SABINENE	0,02
16	16,1	β-MYRCENE	0,05
17	18,4	LIMONENE	0,08
18	19,0	1,8-CINEOLE	0,31
19	20,4	Cis-β-OCIMENE	0,01
20	21,3	γ-TERPINENE	0,01
21	21,5	Trans-β-OCIMENE	0,19
22	22,8	p-CYMENE	0,02
23	24,1	TERPINOLENE	0,01
24	25,7	ACETATE DE cis-3-HEXENYLE	0,01
25	27,3	6-METHYL-5-HEPTEN-2-ONE	0,11
26	30,1	3-HEXEN-1-OL	0,02
27	31,5	FENCHONE	0,01
28	34,3	Cis-OXYDE DE LINALOL	0,03
29	34,4	1-OCTEN-3-OL	0,01
30	35,8	DIHYDROMYRCENOL	0,03
31	36,2	MENTHONE	0,12
32	36,5	ACETATE D'OCTYLE	0,04
33	37,3	ISOMENTHONE	0,02
34	38,0	α-COPAENE	0,07
35	38,9	DECANAL	0,02
36	39,8	CAMPHRE	0,02
37	40,4	β-BOURBONENE	0,03
38	40,6	α-GURJUNENE	0,01
39	41,1	**LINALOL**	**19,32**
40	41,6	1-OCTANOL	0,08
41	41,9	ALCOOL ALIPHATIQUE	0,01
42	42,4	ACETATE DE MENTHYLE	0,03
43	42,8	α,cis-BERGAMOTENE	0,01
44	43,9	α-trans-BERGAMOTENE	0,64
45	44,3	β-ELEMENE	0,06
46	44,6	β-CUBEBENE	0,02
47	44,9	TERPINENE-4-OL	0,03

Tableau de résultats 2 : BASILIC BIO

LOT N° OF22779

Pics	Temps de rétention	Constituants	%
48	45,0	β-CARYOPHYLLENE	0,55
49	47,2	MENTHOL	0,44
[illegible]	[illegible]	[illegible]	[illegible]
54	49,7	α-HUMULENE	0,23
55	50,0	NERAL	0,51
56	50,4	Z-β-FARNESENE	0,01
57	50,7	α-TERPINEOL	0,16
58	51,9	GERMACRENE D	0,42
59	52,1	SESQUITERPENE	0,08
60	52,2	α-ZINGIBERENE	0,03
61	52,3	β-SELINENE	0,01
62	52,6	β-BISABOLENE	0,13
63	52,8	SESQUITERPENE	0,02
64	53,0	GERANIAL	0,61
65	53,1	CARVONE	0,05
66	53,3	BICYCLOGERMACRENE	0,08
67	54,3	ACETATE DE GERANYLE	0,01
68	54,5	δ-CADINENE	0,04
69	54,8	γ-CADINENE	0,01
71	55,3	α-CURCUMENE	0,03
72	55,4	α-BISABOLENE	1,91
73	56,5	NEROL	0,07
74	58,6	Trans-ANETHOL	0,02
75	59,1	GERANIOL	0,06
76	64,5	2,6-DIMETHYL, 3-7-OCTADIENE-2,6-DIOL	0,02
77	67,9	OXYDE DE CARYOPHYLLENE	0,03
78	68,4	METHYLEUGENOL	0,02
79	69,2	ANISALDEHYDE	0,01
80	69,8	NEROLIDOL	0,08
81	70,6	Epoxy-6,7-HUMULENE	0,01
82	70,9	COMPOSÉ AROMATIQUE	0,01
83	74,7	SPATHULENOL	0,02
84	76,4	EUGENOL	0,09
85	78,0	T-CADINOL	0,01
86	78,4	α-MUUROLOL	0,01
87	79,0	α-BISABOLOL	0,03
88	80,1	α-CADINOL	0,01
89	84,4	CHAVICOL	0,03
90	86,6	COMPOSÉ AROMATIQUE Mw=220	0,01
91	95,3	3-METHOXY-CINNAMALDEHYDE	0,08
92	101,3	COMPOSÉ AROMATIQUE Mw=164	0,05
		**TOTAL**	**99,97**

Date de l'analyse – date of the analysis : Avril 2016,

Pranarôm Int.
C. Schulze
Contrôle qualité

Figure S2 (page 4/4) – Certificate of the chemical analysis for the lot number OF22779 of *Ocimum basilicum ssp basilicum* L. (basil) provided by Pranarôm. Screen capture made on 1st September 2020.

PRANARŌM international

FICHE D'ANALYSE – ANALYSIS SHEET

Huile essentielle – Essential oil

Nom botanique – botanical name : ***Matricaria recutita***
Nom commun – french name : MATRICAIRE
Numéro du lot – lot number : **OF22830**
Origine - origin : --- PRANAROM - UK
Partie de la plante – part of the plant : FLEUR
Date de distillation – distillation date : 06/2015
Date de péremption – out of date : 04/2021

Caractéristiques d'analyse - analysis characteristics:

CPG - SM HEWLETT PACKARD
Colonne : HP INNOWAX 60-0.5-0.25
[illegible]

Figure S3 (page 1/6) – Certificate of the chemical analysis for the lot number OF22830 of *Matricaria recutita* L. (German chamomile) provided by Pranarôm. Screen capture made on 1st September 2020.

Aspect – physical state	Liquide limpide
Couleur - colour	Bleu foncé
Odeur - odour	Aromatique, tenace
Densité à 20°C - density	0,895
Densité à 15°C - density	0,899
Indice de réfraction à 20°C – refractive index	1,497 3 (lecture floue)
Pouvoir rotatoire à 20°C - optical rotation	Trop foncé pour la lecture
Miscibilité à l'éthanol à 80% - miscibility	Soluble dans l'alcool à 90 %
Point d'éclair : SETAFLASH - flashpoint	92,2 °C

Analyses pesticides – pesticide analysis :

Pesticides Organochlorés : **Dosage par GC MS détecteur XSD (méthode multirésidus interne validée selon la norme NF V03-110)**	**Résultats**
**Liste des pesticides recherchés (Pharmacopée Européenne)**: Alachlor, Aldrine, Bromophos Ethyl, Bromophos Methyl, Chlordane, Chlorfenvinphos, Chlorpyriphos, Chlorpyriphos Methyl, Chlorthal Dimethyl, Cyfluthrine, Cyhalothrine lambda, Cypermethrine, Dichlofluanide, Dichlorvos, Dicofol (Kelthane), Dieldrine, Endosulfan, Endosulfan sulfate, Endrine, Fenchlorphos (Ronnel), Fenchlorphos-oxon, Fenvalerate, Fluvalinate, Heptachlor, Heptachlor epoxide, Hexachlorobenzene, Hexachlorocyclohexane α, Hexachlorocyclohexane β, Hexachlorocyclohexane δ, Hexachlorocyclohexane ε, Lindane, Methoxychlore, Mirex, Naled, o,p'-DDD , o,p'-DDE, o,p'-DDT, Oxychlordane, p,p'-DDD, p,p'-DDE, p,p'-DDT, Pentachloroaniline, Pentachloroanisole, Permethrine , Phosalone, Procymidone, Profenophos, Prothiofos, Quintozene, S421, Tecnazene, Tetradifon, Vinclozoline	< LMR*<br>* Limite Maximale de Résidus autorisée
Pesticides Organophosphorés : **Dosage par GC MS détecteur FPD (méthode multirésidus interne validée selon la norme NF V03-110)**	**Résultats**
**Liste des pesticides recherchés (Pharmacopée Européenne)**: Acephate, Azinphos Ethyl, Azinphos Méthyl, Bromophos Ethyl, Bromophos Méthyl, Chlorfenvinphos, Chlorpyriphos, Chlorpyriphos Methyl, Diazinon, Dichlorvos, Dimethoate, Ethion, Etrimphos, Fenchlorphos (Ronnel), Fenchlorphos-oxon, Fenitrothion, Fensulfothion (Dasanit), Fensulfothion-oxon, Fensulfothion-oxon-sulfone, Fensulfothion-sulfone, Fenthion, Fenthion-oxon, Fenthion-oxon-sulfone, Fenthion-oxon-sulfoxyde, Fenthion-sulfone, Fenthion-sulfoxyde, Fonofos, Malaoxon, Malathion, Mecarbam, Methacrifos, Methamidophos (Monitor), Methidathion, Monocrotophos, Naled, Omethoate, Paraoxon, Paraoxon Méthyl, Parathion Ethyl, Parathion Methyl, Phosalone, Phosmet, Pirimiphos Ethyl, Pirimiphos Méthyl, Profenophos, Prothiofos, Quinalphos.	< LMR*<br>* Limite Maximale de Résidus autorisée

Profil CHROMATOGRAPHIQUE

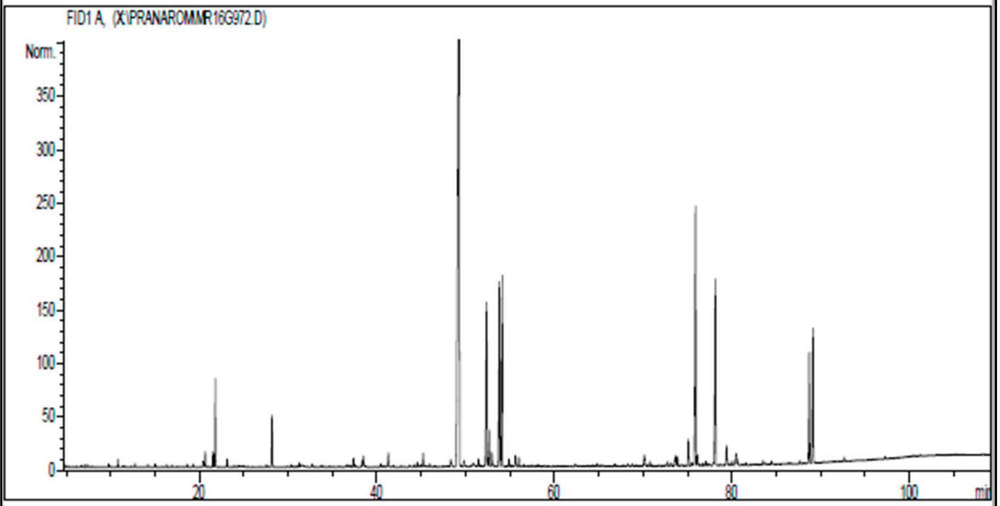

Figure S3 (page 2/6) – Certificate of the chemical analysis for the lot number OF22830 of *Matricaria recutita* L. (German chamomile) provided by Pranarôm. Screen capture made on 1st September 2020.

Tableau de résultats 1 : CAMOMILLE MATRICAIRE

LOT OF22830

Pics	Temps de rétention	Constituants	%
1	4,9	ACETONE	0,02
2	6,2	NONANE	0,01
3	6,6	2-METHYL BUTANAL	0,01
4	6,7	ISOVALERALDEHYDE	0,02
5	7,1	ETHANOL	0,03
6	7,5	2-METHYL BUTANAL	0,03
7	9,8	α-PINENE	0,06
8	9,9	α-THUYENE	0,01
9	10,8	2-METHYLBUTYRATE D'ETHYLE	0,12
10	11,3	VALERATE D'ETHYLE	0,01
11	11,5	CAMPHENE	0,02
12	11,7	ESTER ALIPHATIQUE	0,02
13	12,3	HEXANAL	0,01
14	12,7	UNDECANE	0,07
15	14,2	SABINENE	0,05
16	15,0	METHYLPROPIONATE D'ISOBUTYLE	0,08
17	16,3	β-MYRCENE	0,04
18	16,9	ψ-LIMONENE	0,03
19	17,5	α-TERPINENE	0,01
20	18,7	LIMONENE	0,06
21	19,3	1,8-CINEOLE + β-PHELLANDRENE	0,07
22	20,4	2-PENTYL FURANE	0,12
23	20,5	2-METHYLBUTYRATE DE BUTYLE	0,04
24	20,7	Cis-β-OCIMENE	0,34
25	21,5	γ-TERPINENE	0,35
26	21,8	Trans-β-OCIMENE	1,98
27	23,1	p-CYMENE	0,18
28	24,0	TERPINOLENE	0,04
29	24,4	OCTANAL	0,11
30	25,0	ISOVALERATE D'ISOAMYLE	0,03
31	25,2	4,8-DIMETHYL-1,3,7-NONATRIENE	0,02
32	27,5	6-METHYL-5-HEPTEN-2-ONE	0,06
33	28,2	ARTEMISIA CETONE	1,25
34	30,3	3-HEXEN-1-OL	0,06
35	30,8	PROPIONATE D'HEXENYLE	0,02
36	31,0	ANGELATE DE METHALLYLE	0,02
37	31,3	YOMOGI ALCOOL	0,11
38	31,6	NONANAL	0,18
39	32,7	BUTYRATE D'HEXYLE	0,09
40	33,8	2-METHYLBUTYRATE D'HEXYLE	0,05
41	34,7	3-METHYLBUTYRATE D'HEXYLE	0,03
42	35,1	ACETATE D'ARTEMISYLE ISOMERE	[illegible]

Figure S3 (page 3/6) – Certificate of the chemical analysis for the lot number OF22830 of *Matricaria recutita* L. (German chamomile) provided by Pranarôm. Screen capture made on 1st September 2020.

Tableau de résultats 2 : CAMOMILLE MATRICAIRE

LOT OF22830

Pics	Temps de rétention	Constituants	%
46	37,0	SESQUITERPENE	0,05
47	37,4	BICYCLOELEMENE	0,25
48	37,6	ISOVALERATE DE Cis-3-HEXENYLE	0,04
49	38,0	CYCLOSATIVENE	0,01
50	38,4	α-COPAENE	0,32
51	38,5	ARTEMISIA ALCOOL	0,17
52	40,4	MODHEPHENE	0,09
53	40,8	α-GURJUNENE	0,03
54	41,2	α-ISOCOMENE	0,49
55	41,3	LINALOL	0,04
56	41,6	β1-CUBEBENE	0,02
57	41,9	1-OCTANOL	0,04
58	42,8	CITRONNELLATE DE METHYLE	0,03
59	43,7	ε-CADINENE	0,07
60	44,2	β-ISOCOMENE	0,07
61	44,6	β-ELEMENE	0,14
62	44,9	β-CUBEBENE	0,09
63	45,2	β-CARYOPHYLLENE	0,44
64	45,5	SESQUITERPENE	0,05
65	46,0	AROMADENDRENE	0,11
66	46,4	SESQUITERPENE	0,02
67	46,6	SESQUITERPENE	0,04
68	47,4	CADINA-3,5-DIENE	0,01
69	47,6	4,8-DIMETHYL, 3,7-NONANEDIEN-2-ONE	0,03
70	48,1	CADINENE ISOMERE	0,06
71	48,3	FARNESENE ISOMERE + ALLO-AROMADENDRENE	0,33
72	49,3	**E(trans)-β-FARNESENE**	**41,17**
73	49,8	α-HUMULENE	0,24
74	50,0	γ-SELINENE	0,02
75	50,7	Z-β-FARNESENE	0,07
76	50,9	γ-CURCUMENE	0,18
77	51,0	BORNEOL	0,06
78	51,5	LEDENE	0,27
79	51,7	SESQUITERPENE	0,06
80	52,4	**GERMACRENE D**	**5,46**
81	52,6	Z,E-α-FARNESENE	1,06
82	53,0	β-BISABOLENE	0,49
83	53,8	**BICYCLOGERMACRENE**	**5,89**
84	54,1	**E,E-α-FARNESENE**	**5,57**
85	54,6	ACETATE DE GERANYLE	0,05
86	54,9	δ-CADINENE	0,22
87	55,2	γ-CADINENE	0,06
88	55,4	SALICYLATE DE METHYLE	0,20
89	55,6	β-SESQUIPHELLANDRENE	0,28
90	56,0	α-CURCUMENE	0,20

Figure S3 (page 4/6) – Certificate of the chemical analysis for the lot number OF22830 of *Matricaria recutita* L. (German chamomile) provided by Pranarôm. Screen capture made on 1st September 2020.

Figure S3 (page 5/6) – Certificate of the chemical analysis for the lot number OF22830 of *Matricaria recutita* L. (German chamomile) provided by Pranarôm. Screen capture made on 1st September 2020.

Tableau de résultats 3 : CAMOMILLE MATRICAIRE

LOT OF22830

Pics	Temps de rétention	Constituants	%
91	56,5	SESQUITERPENE Mw=202	0,09
92	57,0	CADINA-1,4-DIENE	0,06
93	57,5	NEROL	0,08
94	57,8	α-AMORPHENE	0,06
95	58,1	SESQUITERPENE Mw=202	0,08
96	58,9	2,4-DECADIENAL	0,06
97	59,8	CALAMENENE	0,04
98	62,3	SESQUIROSEFURANE	0,07
99	62,5	Epi-CUBEBOL	0,05
100	62,8	COMPOSÉ AROMATIQUE	0,04
101	63,3	α-CALACORENE	0,02
102	63,8	DIHYDROCHAMAZULENE ISOMERE	0,05
103	64,8	PHENYLACETONITRILE	0,04
104	64,4	NONADECANE	0,07
105	64,8	DENDROLASINE	0,10
106	65,1	IONONE ISOMERE	0,05
107	65,4	Trans-JASMONE	0,06
108	66,8	COMPOSÉ AROMATIQUE	0,06
109	67,1	SESQUITERPENE	0,02
110	67,8	COMPOSÉ PHENOLIQUE	0,04
111	68,3	BOURBONANOL ISOMERE	0,11
112	68,8	ESTER ALIPHATIQUE	0,08
113	69,3	COMPOSÉ SESQUITERPENIQUE	0,02
114	69,6	ESTER TERPENIQUE	0,02
115	70,1	Trans-NEROLIDOL + ALCOOL ALIPHATIQUE	0,51
116	70,5	ACIDE CAPRYLIQUE	0,06
117	70,8	GLEENOL	0,12
118	71,7	Epi-CUBENOL	0,04
119	71,9	LEVOJUNENOL	0,05
120	72,3	SESQUIPHELLANDROL ISOMERE	0,02
121	72,5	CUBENOL	0,04
122	72,8	GLOBULOL	0,12
123	73,2	VIRIDIFLOROL	0,12
124	73,6	ALCOOL ALIPHATIQUE	0,14
125	73,7	SESQUITERPENOL	0,26
126	73,9	COMPOSÉ TERPENIQUE	0,27
127	74,3	EUDESMOL ISOMERE	0,05
128	75,1	SPATHULENOL + TRIMETHYL PENTADECANONE	0,81
129	75,5	OXYDE DE BISABOLOL C	0,12
130	75,9	**OXYDE B DE α-BISABOLOL**	**8,77**
131	76,2	COMPOSÉ AROMATIQUE Mw=236	0,32
132	76,4	DIHYDROCHAMAZULENE ISOMERE	0,05
133	76,8	EUGENOL	0,07
134	77,1	OXYDE DE BISABOLOL ISOMERE	0,14
135	77,4	T-CADINOL	0,10

Tableau de résultats 4 : CAMOMILLE MATRICAIRE

LOT OF22830

Pics	Temps de rétention	Constituants	%
136	78,0	**OXYDE A DE BISABOLOL**	**5,96**
[illegible]	[illegible]	[illegible]	[illegible]
141	80,4	α-CADINOL + OXYDE DE BISABOLOL ISOMERE	0,14
142	80,5	α-EUDESMOL	0,34
143	80,6	SESQUITERPENOL	0,16
144	81,6	ACIDE CAPRIQUE	0,05
145	83,5	MATRICARIA ESTER	0,13
146	84,4	TRICOSANE	0,09
147	87,6	COMPOSÉ AROMATIQUE Mw=234	0,09
148	88,7	**CHAMAZULENE**	**3,45**
149	89,1	**OXYDE A DE α-BISABOLOL**	**4,02**
150	92,6	PENTACOSANE	0,13
151	97,2	PHYTOL	0,06
152	100,4	ACIDE MYRISTIQUE	0,03
153	101,3	HEPTACOSANE	0,03
		**TOTAL**	**99,74**

Date de l'analyse – date of the analysis : Avril 2016,

Pranarôm Int.
C. Schulze
Contrôle qualité

Figure S3 (page 6/6) – Certificate of the chemical analysis for the lot number OF22830 of *Matricaria recutita* L. (German chamomile) provided by Pranarôm. Screen capture made on 1st September 2020.

PRANARÔM
international

FICHE D'ANALYSE – ANALYSIS SHEET

Huile essentielle – Essential oil

Nom botanique – botanical name : *Cinnamomum cassia*
Nom commun – french name : CANNELIER DE CHINE
Numéro du lot – lot number : **OF22426**
Origine - origin : --- PRANARÔM - CHINE
Partie de la plante – part of the plant : RAMEAU
Date de distillation – distillation date : 06/2015
Date de péremption – out of date : 04/2021

Caractéristiques d'analyse- analysis characteristics:

CPG - SM HEWLETT PACKARD / CPG-FID

Figure S4 (page 1/4) – Certificate of the chemical analysis for the lot number OF22426 of *Cinnamomum cassia* (L.) J. Presl. (Chinese cinnamon) provided by Pranarôm. Screen capture made on 1st September 2020.

Aspect – physical state	Liquide limpide
Couleur - colour	Jaune clair
Odeur - odour	Caractéristique, de cinnamaldehyde
Densité à 20°C - density	1,051
Densité à 15°C - density	1,055
Indice de réfraction à 20°C - refractive index	1,609 8
Pouvoir rotatoire à 20°C - optical rotation	0 °
Miscibilité à l'éthanol à 70% - miscibility	1,9 volumes d'alcool / 1 volume d'HE
Point d'éclair : SETAFLASH - flashpoint	113,1 °C

Analyses pesticides – pesticide analysis :

**Pesticides Organochlorés : Dosage par GC MS détecteur XSD (méthode multirésidus interne validée selon la norme NF V03-110)** **Liste des pesticides recherchés (Pharmacopée Européenne)**: Alachlor, Aldrine, Bromophos Ethyl, Bromophos Methyl, Chlordane, Chlorfenvinphos, Chlorpyriphos, Chlorpyriphos Methyl, Chlorthal Dimethyl, Cyfluthrine, Cyhalothrine lambda, Cypermethrine, Dichlofluanide, Dichlorvos, Dicofol (Kelthane), Dieldrine, Endosulfan, Endosulfan sulfate, Endrine, Fenchlorphos (Ronnel), Fenchlorphos-oxon, Fenvalerate, Fluvalinate, Heptachlor, Heptachlor epoxide, Hexachlorobenzene, Hexachlorocyclohexane α, Hexachlorocyclohexane β, Hexachlorocyclohexane δ, Hexachlorocyclohexane ε, Lindane, Methoxychlore, Mirex, Naled, o,p'-DDD , o,p'-DDE, o,p'-DDT, Oxychlordane, p,p'-DDD, p,p'-DDE, p,p'-DDT, Pentachloroaniline, Pentachloroanisole, Permethrine , Phosalone, Procymidone, Profenophos, Prothiofos, Quintozene, S421, Tecnazene, Tetradifon, Vinclozoline	**Résultats** < LMR* * Limite Maximale de Résidus autorisée
**Pesticides Organophosphorés : Dosage par GC MS détecteur FPD (méthode multirésidus interne validée selon la norme NF V03-110)** **Liste des pesticides recherchés (Pharmacopée Européenne)**: Acephate, Azinphos Ethyl, Azinphos Méthyl, Bromophos Ethyl, Bromophos Méthyl, Chlorfenvinphos, Chlorpyriphos, Chlorpyriphos Methyl, Diazinon, Dichlorvos, Dimethoate, Ethion, Etrimphos, Fenchlorphos (Ronnel), Fenchlorphos-oxon, Fenitrothion, Fensulfothion (Dasanit), Fensulfothion-oxon, Fensulfothion-oxon-sulfone, Fensulfothion-sulfone, Fenthion, Fenthion-oxon, Fenthion-oxon-sulfone, Fenthion-oxon-sulfoxyde, Fenthion-sulfone, Fenthion-sulfoxyde, Fonofos, Malaoxon, Malathion, Mecarbam, Methacrifos, Methamidophos (Monitor), Methidathion, Monocrotophos, Naled, Omethoate, Paraoxon, Paraoxon Méthyl, Parathion Ethyl, Parathion Methyl, Phosalone, Phosmet, Pirimiphos Ethyl, Pirimiphos Méthyl, Profenophos, Prothiofos, Quinalphos.	**Résultats** < LMR* * Limite Maximale de Résidus autorisée

Profil CHROMATOGRAPHIQUE

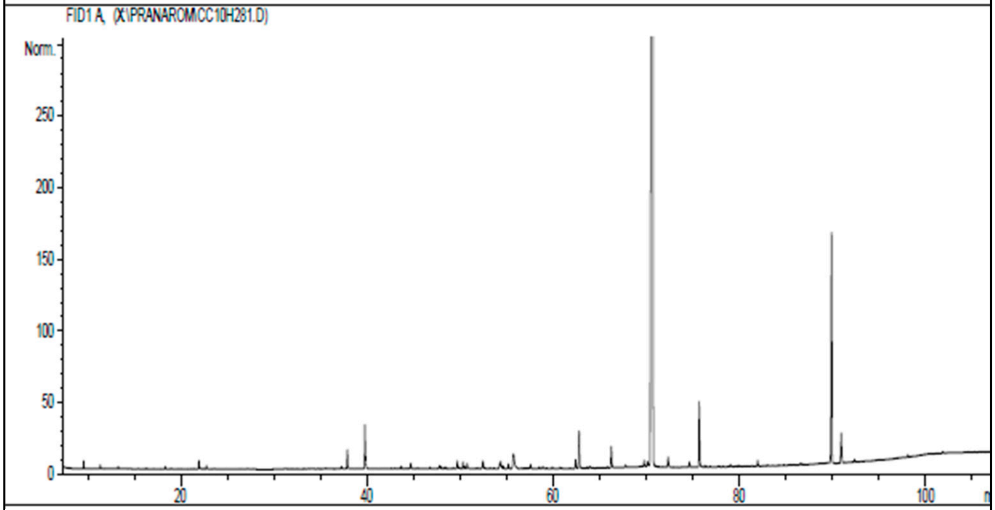

Figure S4 (page 2/4) – Certificate of the chemical analysis for the lot number OF22426 of *Cinnamomum cassia* (L.) J. Presl. (Chinese cinnamon) provided by Pranarôm. Screen capture made on 1st September 2020.

Tableau de résultats 1 : CINNAMOMUM CASSIA

OF22426

Pics	Temps de rétention	Constituants	%
1	5,0	ACETONE	0,01
2	6,0	METHANOL	0,04
3	9,5	α-PINENE	0,09
4	11,3	CAMPHENE	0,06
5	13,2	β-PINENE	0,04
6	16,3	α-PHELLANDRENE	0,01
7	18,3	LIMONENE	0,04
8	18,9	β-PHELLANDRENE + 1,8-CINEOLE	0,01
9	21,9	STYRENE	0,16
10	22,7	p-CYMENE	0,06
11	27,1	6-METHYL-5-HEPTEN-2-ONE	0,02
12	35,5	FURFURALDEHYDE	0,02
13	37,2	YLANGENE	0,06
14	37,8	α-COPAENE	0,41
15	39,7	BENZALDEHYDE	0,99
16	42,6	α-cis-BERGAMOTENE	0,02
17	43,0	SESQUITERPENE	0,04
18	43,4	ACETATE DE BORNYLE	0,02
19	43,6	α-trans-BERGAMOTENE	0,05
20	44,0	β-ELEMENE	0,01
21	44,4	TERPINENE-4-OL	0,02
22	44,7	β-CARYOPHYLLENE	0,12
23	45,4	AROMADENDRENE	0,02
24	46,7	p-METHOXY STYRENE	0,03
25	47,7	ACETOPHENONE	0,06
[illegible]	[illegible]	[illegible]	[illegible]
31	49,8	α-HUMULENE	0,03
32	50,3	γ-MUUROLENE + BORNEOL	0,13
33	50,5	LEDENE	0,04
34	50,7	β-BISABOLENE	0,10
35	50,8	HYDROXY CINNAMALDEHYDE	0,02
36	52,4	α-MUUROLENE	0,22
37	52,6	SESQUITERPENE	0,01
38	54,2	SESQUITERPENE	0,09
39	54,3	δ-CADINENE	0,20
40	54,6	FARNESENE ISOMERE	0,06
41	55,2	γ-CADINENE	0,09
42	55,7	BENZENE PROPANAL + α-CURCUMENE	0,71
43	56,6	SESQUITERPENE	0,02
44	57,4	SESQUITERPENE	0,02
45	57,5	ACETATE DE 2-PHENYLETHYLE	0,07

Figure S4 (page 3/4) – Certificate of the chemical analysis for the lot number OF22426 of *Cinnamomum cassia* (L.) J. Presl. (Chinese cinnamon) provided by Pranarôm. Screen capture made on 1st September 2020.

Tableau de résultats 2 : CINNAMOMUM CASSIA

OF22426

Pics	Temps de rétention	Constituants	%
46	58,4	Trans-ANETHOL	0,03
47	58,9	CALAMENENE	0,05
48	59,3	2-METHOXY PHENOL	0,01
49	59,9	ALCOOL BENZYLIQUE	0,03
50	60,7	COMPOSÉ CINNAMALDEHYDE	0,02
51	62,4	Z-CINNAMALDEHYDE	0,19
52	62,7	ALCOOL PHENYLETHYLIQUE	0,76
53	63,6	α-CALACORENE	0,02
54	63,9	TETRADECANAL	0,04
55	65,7	COMPOSÉ PHENOLIQUE	0,02
56	66,2	2-METHOXY-BENZALDEHYDE	0,46
57	67,7	OXYDE DE CARYOPHYLLENE	0,05
58	69,7	E-NEROLIDOL	0,12
59	70,1	3-PHENYLPROPANOL	0,13
60	70,6	**E-CINNAMALDEHYDE**	**84,96**
61	72,3	COMPOSÉ PHENYLETHYLIQUE Mw=164	0,27
62	74,6	SPATHULENOL	0,11
63	75,0	ACETATE DE CINNAMYLE	1,29
64	76,2	EUGENOL	0,03
65	76,4	α-MUUROLOL	0,02
66	76,8	EPOXY SESQUITERPENIQUE	0,02
67	77,6	δ-CADINOL	0,02
68	77,8	SESQUITERPENOL	0,02
69	78,1	EXPOXY SESQUITERPENIQUE	0,02
70	79,0	DITERPENE Mw=272	0,05
71	79,5	CADALENE	0,02
72	79,6	α-CADINOL	0,02
73	79,8	SESQUITERPENOL	0,02
74	80,0	COMPOSÉ AROMATIQUE	0,02
75	81,3	2-METHOXY CINNAMALDEHYDE	0,01
76	81,9	ALCOOL CINNAMIQUE	0,12
82	90,9	COUMARINE Mw=146	0,75
83	92,3	ACETATE D'o-METHOXYCINNAMALDEHYDE	0,05
84	98,0	BENZOATE DE BENZYLE	0,05
85	101,8	BENZOATE DE 2-PHENYLETHYLE	0,04
86	107,8	ACIDE CINNAMIQUE	0,11
		**TOTAL**	**99,94**

Figure S4 (page 4/4) – Certificate of the chemical analysis for the lot number OF22426 of *Cinnamomum cassia* (L.) J. Presl. (Chinese cinnamon) provided by Pranarôm. Screen capture made on 1st September 2020.

Date de l'analyse – date of the analysis : Mai 2016,

Pranarôm Int.
C. Schulze
Contrôle qualité

PRANARŌM international

FICHE D'ANALYSE – ANALYSIS SHEET

Huile essentielle – Essential oil

Nom botanique – botanical name: ***Coriandrum sativum***
Nom commun – french name: CORIANDRE
Numéro du lot – lot number : **OF21374**
Origine - origin: ---- PRANARÔM - UK
Partie de la plante – part of the plant: FEUILLE
Date de distillation – distillation date : 06/2014
Date de péremption – out of date : 12/2019

Caractéristiques d'analyse – analysis characteristics:

CPG - SM HEWLETT PACKARD / CPG-FID
Colonne : HP INNOWAX 60-0.5-0.25
Programmation de température : 6 mn à 50 °C –2 °C/mn→250 °C-10mn à 250 °C
Gaz vecteur He : 22 psis

Caractéristiques physiques – physical characteristics:

Aspect – physical state	Liquide limpide
Couleur - colour	Jaune clair
Odeur - odour	Caractéristique de la plante
Densité à 20°C - density	0,860
Densité à 15°C - density	0,864
Indice de réfraction à 20°C - refractive index	1,457 2
Pouvoir rotatoire à 20°C - optical rotation	+ 5 °
Miscibilité à l'éthanol à 75% - miscibility	1,3 volumes d'alcool / 1 volume d'HE
Point d'éclair : SETAFLASH - flashpoint	70,4 °C

Analyses pesticides – pesticide analysis :

Pesticides Organochlorés : Dosage par GC MS détecteur XSD (méthode multirésidus interne validée selon la norme NF V03-110)	Résultats
**Liste des pesticides recherchés (Pharmacopée Européenne):** Alachlor, Aldrine, Bromophos Ethyl, Bromophos Methyl, Chlordane, Chlorfenvinphos, Chlorpyriphos, Chlorpyriphos Methyl, Chlorthal Dimethyl, Cyfluthrine, Cyhalothrine lambda, Cypermethrine, Dichlofluanide, Dichlorvos, Dicofol (Kelthane), Dieldrine, Endosulfan, Endosulfan sulfate, Endrine, Fenchlorphos (Ronnel), Fenchlorphos-oxon, Fenvalerate, Fluvalinate, Heptachlor, Heptachlor epoxide, Hexachlorobenzene, Hexachlorocyclohexane α, Hexachlorocyclohexane β, Hexachlorocyclohexane δ, Hexachlorocyclohexane ε, Lindane, Methoxychlore, Mirex, Naled, o,p'-DDD , o,p'-DDE, o,p'-DDT, Oxychlordane, p,p'-DDD, p,p'-DDE, p,p'-DDT, Pentachloroaniline, Pentachloroanisole, Permethrine , Phosalone, Procymidone, Profenophos, Prothiofos, Quintozene, S421, Tecnazene, Tetradifon, Vinclozoline	< LMR* * Limite Maximale de Résidus autorisée

Figure S5 (page 1/5) – Certificate of the chemical analysis for the lot number OF21374 of *Coriandrum sativum* L. (coriander) provided by Pranarôm. Screen capture made on 1st September 2020.

sulfone, Fenthion-oxon-sulfoxyde, Fenthion-sulfone, Fenthion-sulfoxyde, Fonofos, Malaoxon, Malathion, Mecarbam, Methacrifos, Methamidophos (Monitor), Methidathion, Monocrotophos, Naled, Omethoate, Paraoxon, Paraoxon Méthyl, Parathion Ethyl, Parathion Methyl, Phosalone, Phosmet, Pirimiphos Ethyl, Pirimiphos Méthyl, Profenophos, Prothiofos, Quinalphos.	* Limite Maximale de Résidus autorisée

Profil CHROMATOGRAPHIQUE

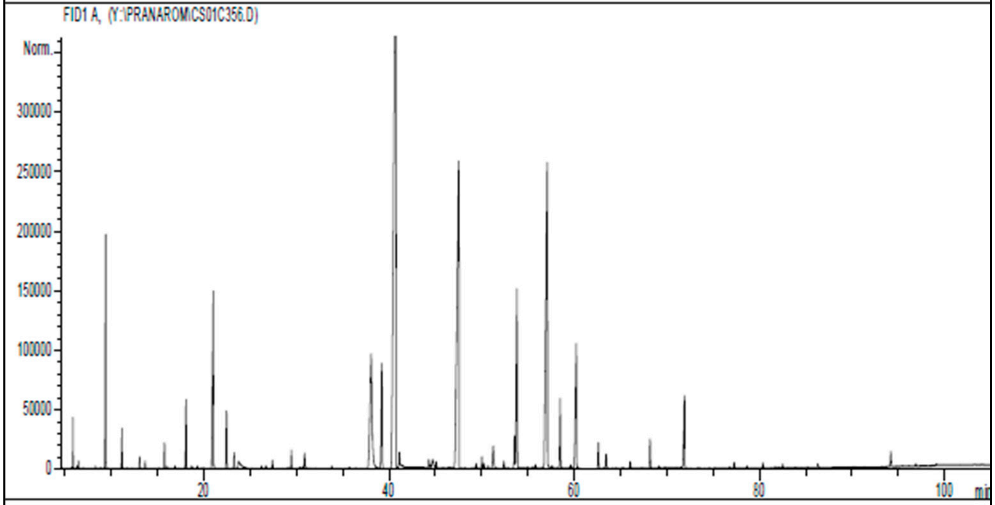

Figure S5 (page 2/5) – Certificate of the chemical analysis for the lot number OF21374 of *Coriandrum sativum* L. (coriander) provided by Pranarôm. Screen capture made on 1st September 2020.

Tableau de résultats 1 : CORIANDRUM SATIVUM

OF21374

Pics	Temps de rétention	Constituants	%
1	4,6	ACETONE	0,02
2	5,7	METHANOL	0,01
3	5,8	NONANE	0,39
4	6,2	2-METHYLBUTANAL	0,01
5	6,3	ISOVALERALDEHYDE	0,03
6	6,4	ETHANOL	0,07
7	6,8	1-NONENE	0,01
8	7,0	2-ETHYL FURANE	0,01
9	8,3	DECANE	0,03
10	8,7	2-METHYLBUTYRATE DE METHYLE	0,01
11	8,8	TRICYCLENE	0,02
12	9,3	**α-PINENE**	**3,28**
13	9,4	α-THUYENE	0,01
14	11,1	CAMPHENE	0,55
15	11,7	HEXANAL	0,01
16	12,3	UNDECANE	0,01
17	13,0	β-PINENE	0,17
18	13,6	SABINENE	0,10
19	15,1	Δ3-CARENE	0,01
20	15,7	β-MYRCENE	0,36
21	16,0	α-PHELLANDRENE	0,03
22	16,9	α-TERPINENE	0,04
23	17,2	HEPTANAL	0,01
24	18,0	LIMONENE	1,09
25	18,7	β-PHELLANDRENE	0,04
26	18,8	1,8-CINEOLE	0,02
27	19,3	2-HEXANAL	0,04
28	19,7	2-PENTYLFURANE	0,01
29	20,0	Cis-β-OCIMENE	0,01
30	21,0	**γ-TERPINENE**	**4,14**
31	21,1	Trans-β-OCIMENE	0,05
32	22,4	p-CYMENE	0,90
33	23,3	TERPINOLENE	0,25
34	23,7	OCTANAL	0,65
35	25,2	ACETATE DE 4-HEXEN-1-OL	0,01
36	26,2	2-HEXYLFURANE	0,04
37	26,6	6-METHYL-5-HEPTEN-2-ONE	0,06
38	27,4	1-HEXANOL	0,14
39	29,1	ANGELATE DE METHALLYLE	0,02
40	29,4	3-HEXEN-1-OL	0,31
41	30,4	2-NONANONE	0,04
42	30,7	NONANAL	0,05
43	30,9	2-HEXEN-1-OL	0,32
44	32,0	PENTYL BENZENE	0,01
45	33,8	α,p-DIMETHYL STYRENE + Cis-OXYDE DE LINALOL	0,04

Figure S5 (page 3/5) – Certificate of the chemical analysis for the lot number OF21374 of *Coriandrum sativum* L. (coriander) provided by Pranarôm. Screen capture made on 1st September 2020.

Figure S5 (page 4/5) – Certificate of the chemical analysis for the lot number OF21374 of *Coriandrum sativum* L. (coriander) provided by Pranarôm. Screen capture made on 1st September 2020.

Tableau de résultats 2 : CORIANDRUM SATIVUM

OF21374

Pics	Temps de rétention	Constituants	%
46	34,3	6-METHYL-5-HEPTEN-2-OL	0,01
47	35,1	Trans-THUYANOL	0,01
48	35,7	Trans-OXYDE DE LINALOL	0,03
49	35,8	ACETATE D'OCTYLE	0,01
50	36,4	CITRONELLAL	0,03
51	38,1	**DECANAL**	**6,43**
52	39,2	CAMPHRE	2,40
53	40,7	**LINALOL**	**32,42**
54	41,0	Cis-THUYANOL	0,10
55	41,1	1-OCTANOL	0,72
56	42,5	PINOCARVONE	0,07
57	44,3	TERPINENE-4-OL	0,20
58	44,4	β-CARYOPHYLLENE	0,07
59	44,7	UNDECANAL	0,42
60	45,1	ALCOOL CYCLOALKYL	0,17
61	45,7	Cis-p-MENTH-2-EN-1-OL	0,03
62	45,9	BENZOATE DE METHYLE	0,03
63	46,3	Cis-β-TERPINEOL	0,03
64	46,5	MYRTENAL	0,06
65	47,5	**2-DECENAL**	**15,09**
66	47,6	1-NONANOL	0,10
67	47,9	ACETATE DE CITRONELLYLE	0,01
68	48,2	ISOBORNEOL	0,02
69	48,9	δ-TERPINEOL	0,01
70	49,1	α-HUMULENE + LACTONE LAVANDE	0,01
71	49,4	NERAL + FORMIATE DE DECYLE	0,10
72	49,7	ACETATE DE MYRTENYLE	0,01
73	50,0	α-TERPINEOL	0,19
74	50,2	BORNEOL	0,09
75	50,6	FORMIATE DE GERANYLE	0,05
76	51,2	DODECANAL	0,67
77	51,3	VERBENONE	0,05
78	51,7	ACETATE DE NERYLE	0,02
79	52,4	GERANIAL	0,14
80	52,6	OXYDE DE LINALOL PYRANIQUE	0,02
81	52,8	CARVONE	0,03
82	53,5	ACETATE DE GERANYLE	0,55
83	53,8	**1-DECANOL + 2-UNDECENAL**	**4,27**
84	54,1	CITRONELLOL	0,03
85	54,4	SESQUITERPENE	0,02
86	54,6	PROPIONATE DE NERYLE	0,02
87	55,0	SALICYLATE DE METHYLE	0,05
88	55,5	MYRTENOL	0,04
89	55,8	NEROL	0,07
90	57,1	**2-DECEN-1-OL**	**12,35**

Figure S5 (page 5/5) – Certificate of the chemical analysis for the lot number OF21374 of *Coriandrum sativum* L. (coriander) provided by Pranarôm. Screen capture made on 1st September 2020.

Tableau de résultats 3 : CORIANDRUM SATIVUM

OF21374

Pics	Temps de rétention	Constituants	%
91	58,2	ALCOOL ALIPHATIQUE	0,06
92	57,6	ALDEHYDE TERPENIQUE	0,05
93	58,0	Trans-CARVEOL	0,01
94	58,4	GERANIOL	1,24
95	58,5	p-CYMENE-8-OL	0,02
96	59,6	ALCOOL ALIPHATIQUE	0,07
97	60,2	**2-DODECENAL**	**3,54**
98	60,4	DODECADIENAL	0,03
99	62,6	2-DODECEN-1-OL	0,45
100	63,4	PENTADECANAL	0,30
101	63,5	2,6-DIMETHYL-3,7-OCTADIENE-2,6-DIOL	0,03
102	65,2	1-DODECANOL	0,02
103	66,0	2-TRIDECENAL	0,13
104	67,3	OXYDE DE CARYOPHYLLENE	0,01
105	68,2	Trans-2-TETRADECEN-1-OL	0,50
106	69,1	Cis-NEROLIDOL	0,06
107	69,8	ACIDE CAPRYLIQUE	0,02
108	71,9	TETRADECENAL	1,81
109	77,3	ALDEHYDE ALIPHATIQUE INSATURÉ	0,10
110	78,6	ALDEHYDE ALIPHATIQUE	0,04
111	80,4	ACIDE CAPRIQUE	0,10
112	81,7	COMPOSÉ PHENYLIQUE Mw=238	0,02
113	82,5	ALDEHYDE ALIPHATIQUE INSATURÉ	0,08
114	86,3	ACIDE trans-2-DECENOÏQUE	0,07
115	94,2	SEDANOLIDE Mw=194	0,32
116	96,9	COMPOSÉ AROMATIQUE	0,04
117	99,1	ACIDE MYRISTIQUE	0,04
		**TOTAL**	**99,84**

Date de l'analyse – date of the analysis : Janvier 2015,

Pranarôm Int.
C. Schulze
Contrôle qualité

Figure S6 (page 1/4) – Certificate of the chemical analysis for the lot number OF22293 of *Eugenia caryophyllus* (Spreng.) Bullock & S. G. Harrison. (clove) provided by Pranarôm. Screen capture made on 1st September 2020.

FICHE D'ANALYSE – ANALYSIS SHEET

Huile essentielle – Essential oil

Nom botanique – botanical name: ***Eugenia caryophyllus***
Nom commun – french name: CLOUS DE GIROFLE
Numéro du lot – lot number : **OF22293**
Origine - origin : -- PRANARÔM - INDONESIE
Partie de la plante – part of the plant: BOUTON FLORAL
Date de distillation – distillation date : 06/2015
Date de péremption – out of date : 04/2021

Caractéristiques d'analyse – analysis characteristics:

CPG - SM HEWLETT PACKARD / CPG - FID
Colonne : HP INNOWAX 60-0.5-0.25
Programmation de température : 6 mn à 50°C -2°C/mn→250°C-20mn à 250°C
Gaz vecteur He : 22 psis

Caractéristiques physiques – physical characteristics :

Aspect – physical state	Liquide limpide
Couleur - colour	Jaune d'or clair
Odeur - odour	Caractéristique de l'eugénol
Densité à 20°C - density	1,055
Densité à 15°C - density	1,059
Indice de réfraction à 20°C - refractive index	1,534 8
Pouvoir rotatoire à 20°C - optical rotation	- 0,5 °
Miscibilité à l'éthanol à 70% - miscibility	1 volume d'alcool / 1 volume d'HE
Point d'éclair : SETAFLASH - flashpoint	103,3 °C

Analyses pesticides – pesticide analysis :

Pesticides Organochlorés : Dosage par GC MS détecteur XSD (méthode multirésidus interne validée selon la norme NF V03-110)	Résultats
**Liste des pesticides recherchés (Pharmacopée Européenne)**: Alachlor, Aldrine, Bromophos Ethyl, Bromophos Methyl, Chlordane, Chlorfenvinphos, Chlorpyriphos, Chlorpyriphos Methyl, Chlorthal Dimethyl, Cyfluthrine, Cyhalothrine lambda, Cypermethrine, Dichlofluanide, Dichlorvos, Dicofol (Kelthane), Dieldrine, Endosulfan, Endosulfan sulfate, Endrine, Fenchlorphos (Ronnel), Fenchlorphos-oxon, Fenvalerate, Fluvalinate, Heptachlor, Heptachlor epoxide, Hexachlorobenzene, Hexachlorocyclohexane α, Hexachlorocyclohexane β, Hexachlorocyclohexane δ, Hexachlorocyclohexane ε, Lindane, Methoxychlore, Mirex, Naled, o,p'-DDD , o,p'-DDE, o,p'-DDT, Oxychlordane, p,p'-DDD, p,p'-DDE, p,p'-DDT, Pentachloroaniline, Pentachloroanisole, Permethrine , Phosalone, Procymidone, Profenophos, Prothiofos, Quintozene, S421, Tecnazene, Tetradifon, Vinclozoline	< LMR*<br>* Limite Maximale de Résidus autorisée
**Pesticides Organophosphorés : Dosage par GC MS détecteur FPD (méthode multirésidus interne validée selon la norme NF V03-110)**	**Résultats**
**Liste des pesticides recherchés (Pharmacopée Européenne)**: Acephate, Azinphos Ethyl, Azinphos Méthyl, Bromophos Ethyl, Bromophos Méthyl, Chlorfenvinphos, Chlorpyriphos, Chlorpyriphos Methyl, Diazinon, Dichlorvos, Dimethoate, Ethion, Etrimphos, Fenchlorphos (Ronnel), Fenchlorphos-oxon, Fenitrothion, Fensulfothion (Dasanit), Fensulfothion-oxon, Fensulfothion-oxon-sulfone, Fensulfothion-sulfone, Fenthion, Fenthion-oxon, Fenthion-oxon-sulfone, Fenthion-oxon-sulfoxyde, Fenthion-sulfone, Fenthion-sulfoxyde, Fonofos, Malaoxon, Malathion, Mecarbam, Methacrifos, Methamidophos (Monitor), Methidathion, Monocrotophos, Naled, Omethoate, Paraoxon, Paraoxon Méthyl, Parathion Ethyl, Parathion Methyl, Phosalone, Phosmet, Pirimiphos Ethyl, Pirimiphos Méthyl, Profenophos, Prothiofos, Quinalphos.	< LMR*<br>* Limite Maximale de Résidus autorisée

Profil CHROMATOGRAPHIQUE

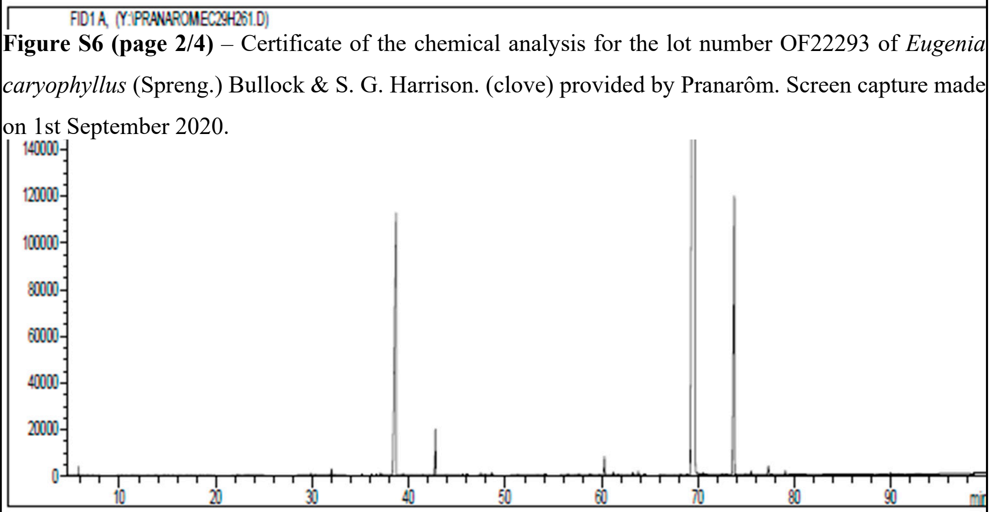

Figure S6 (page 2/4) – Certificate of the chemical analysis for the lot number OF22293 of *Eugenia caryophyllus* (Spreng.) Bullock & S. G. Harrison. (clove) provided by Pranarôm. Screen capture made on 1st September 2020.

Tableau de résultats 1: EUGENIA CARYOPHYLLUS

OF22293

Pics	Temps de rétention	Constituants	%
1	5,8	ETHANOL	0,05
2	22,2	6-METHYL-5-HEPTEN-2-ONE	0,01
3	29,9	FURFURAL	0,02
[illegible]	[illegible]	[illegible]	[illegible]
[illegible]	[illegible]	SESQUITERPENE	[illegible]
9	37,1	ISOCARYOPHYLLENE ISOMERE	0,04
10	38,2	β-ELEMENE	0,01
11	38,7	**β-CARYOPHYLLENE**	**9,06**
12	39,4	SESQUITERPENE	0,02
13	40,5	CADINA-1,5-DIENE	0,01
14	41,5	SESQUITERPENE	0,01
15	42,8	α-HUMULENE	0,70
16	43,9	γ-MUUROLENE	0,01
17	45,6	FARNESENE ISOMERE	0,01
18	46,0	α-MUUROLENE	0,01
19	46,2	β-SELINENE	0,01
20	47,5	α-FARNESENE	0,03
21	47,9	δ-CADINENE	0,01
22	48,0	γ-CADINENE	0,01
23	48,6	SALICYLATE DE METHYLE	0,03
24	51,3	EPOXYDE SESQUITERPENIQUE	0,02
25	54,2	ALCOOL BENZYLIQUE	0,01
26	56,6	α-CALACORENE	0,03
27	57,7	CETONE AROMATIQUE Mw=164	0,03
28	58,8	EPOXYDE SESQUITERPENIQUE	0,02
29	59,0	EPOXYDE SESQUITERPENIQUE	0,01
30	59,8	OXYDE D'ISOCARYOPHYLLENE	0,02
31	60,3	OXYDE DE CARYOPHYLLENE	0,31
32	61,2	EPOXYDE SESQUITERPENIQUE	0,05
33	61,7	METHYLEUGENOL	0,02
34	62,0	EPOXYDE SESQUITERPENIQUE	0,01
35	63,2	EPOXY-6,7-HUMULENE	0,05
36	63,8	CARYOPHYLLENOL	0,06
37	66,1	METHOXY PROPYL PHENOL	0,01
38	66,5	COMPOSÉ AROMATIQUE	0,01
39	68,2	COMPOSÉ OXYGÉNÉ	0,02
40	68,9	EPOXYDE SESQUITERPENIQUE	0,02
41	69,7	**EUGENOL**	**82,30**
42	70,5	SESQUITERPENOL	0,02
43	72,6	COMPOSÉ PHENOLIQUE	0,01
44	73,8	**ACETATE D'EUGENYLE**	**6,40**
45	73,9	COMPOSE Mw=206	0,01

Figure S6 (page 3/4) – Certificate of the chemical analysis for the lot number OF22293 of *Eugenia caryophyllus* (Spreng.) Bullock & S. G. Harrison. (clove) provided by Pranarôm. Screen capture made on 1st September 2020.

Tableau de résultats 2 : EUGENIA CARYOPHYLLUS

OF22293

Pics	Temps de rétention	Constituants	%
46	74,9	SESQUITERPENOL	0,02
47	75,5	CARYOPHYLLA-3,7-DIEN-6-OL	0,06
48	77,2	CHAVICOL	0,10
49	77,3	EPOXYDE SESQUITERPENIQUE	0,09
50	77,6	ISOEUGENOL	0,01
51	77,7	EPOXYDE SESQUITERPENIQUE	0,01
52	79,0	SESQUITERPENOL	0,06
53	87,0	VANILLINE	0,01
54	89,9	BENZOATE DE BENZYLE	0,02
		**TOTAL**	**99,99**

Figure S6 (page 4/4) – Certificate of the chemical analysis for the lot number OF22293 of *Eugenia caryophyllus* (Spreng.) Bullock & S. G. Harrison. (clove) provided by Pranarôm. Screen capture made on 1st September 2020.

PRANARÔM
international

FICHE D'ANALYSE – ANALYSIS SHEET

Huile essentielle – Essential oil

Nom botanique – botanical name : *Cymbopogon citratus*
Nom commun – french name : LEMONGRASS
Numéro du lot – lot number : **OF20407**
Origine - origin: ----- PRANAROM - GUATEMALA
Partie de la plante – part of the plant : PARTIE AÉRIENNE
Date de distillation – distillation date : 06/2014
Date de péremption – out of date : 05/2020

Caractéristiques d'analyse - analysis characteristics:

CPG - SM HEWLETT PACKARD
Colonne : HP INNOWAX 60-0.5-0.25
Programmation de température : 6 mn à 50°C -2°C/mn→250°C-10mn à 250°C

Figure S7 (page 1/5) – Certificate of the chemical analysis for the lot number OF20407 of *Cymbopogon citratus* (DC.) Staph. (lemongrass) provided by Pranarôm. Screen capture made on 1st September 2020.

Couleur - colour	Jaune clair
Odeur - odour	Citronnée caractéristique
Densité à 20°C - density	0,896
Densité à 15°C – density	0,900
Indice de réfraction à 20°C - refractive index	- 0,5 °
Pouvoir rotatoire à 20°C – optical rotation	1,484 4
Miscibilité à l'éthanol à 75% - miscibility	1,2 volumes d'alcool/ 1 volume d'HE
Point d'éclair : SETAFLASH – flashpoint	85,6

Analyses pesticides – pesticide analysis :

	Résultats
**Pesticides Organochlorés : Dosage par GC MS détecteur XSD (méthode multirésidus interne validée selon la norme NF V03-110)** **Liste des pesticides recherchés (Pharmacopée Européenne)**: Alachlor, Aldrine, Bromophos Ethyl, Bromophos Methyl, Chlordane, Chlorfenvinphos, Chlorpyriphos, Chlorpyriphos Methyl, Chlorthal Dimethyl, Cyfluthrine, Cyhalothrine lambda, Cypermethrine, Dichlofluanide, Dichlorvos, Dicofol (Kelthane), Dieldrine, Endosulfan, Endosulfan sulfate, Endrine, Fenchlorphos (Ronnel), Fenchlorphos-oxon, Fenvalerate, Fluvalinate, Heptachlor, Heptachlor epoxide, Hexachlorobenzene, Hexachlorocyclohexane α, Hexachlorocyclohexane β, Hexachlorocyclohexane δ, Hexachlorocyclohexane ε, Lindane, Methoxychlore, Mirex, Naled, o,p'-DDD , o,p'-DDE, o,p'-DDT, Oxychlordane, p,p'-DDD, p,p'-DDE, p,p'-DDT, Pentachloroaniline, Pentachloroanisole, Permethrine , Phosalone, Procymidone, Profenophos, Prothiofos, Quintozene, S421, Tecnazene, Tetradifon, Vinclozoline	< LMR* * Limite Maximale de Résidus autorisée
**Pesticides Organophosphorés : Dosage par GC MS détecteur FPD (méthode multirésidus interne validée selon la norme NF V03-110)** **Liste des pesticides recherchés (Pharmacopée Européenne)**: Acephate, Azinphos Ethyl, Azinphos Méthyl, Bromophos Ethyl, Bromophos Méthyl, Chlorfenvinphos, Chlorpyriphos, Chlorpyriphos Methyl, Diazinon, Dichlorvos, Dimethoate, Ethion, Etrimphos, Fenchlorphos (Ronnel), Fenchlorphos-oxon, Fenitrothion, Fensulfothion (Dasanit), Fensulfothion-oxon, Fensulfothion-oxon-sulfone, Fensulfothion-sulfone, Fenthion, Fenthion-oxon, Fenthion-oxon-sulfone, Fenthion-oxon-sulfoxyde, Fenthion-sulfone, Fenthion-sulfoxyde, Fonofos, Malaoxon, Malathion, Mecarbam, Methacrifos, Methamidophos (Monitor), Methidathion, Monocrotophos, Naled, Omethoate, Paraoxon, Paraoxon Méthyl, Parathion Ethyl, Parathion Methyl, Phosalone, Phosmet, Pirimiphos Ethyl, Pirimiphos Méthyl, Profenophos, Prothiofos, Quinalphos.	**Résultats** < LMR* * Limite Maximale de Résidus autorisée

Profil CHROMATOGRAPHIQUE

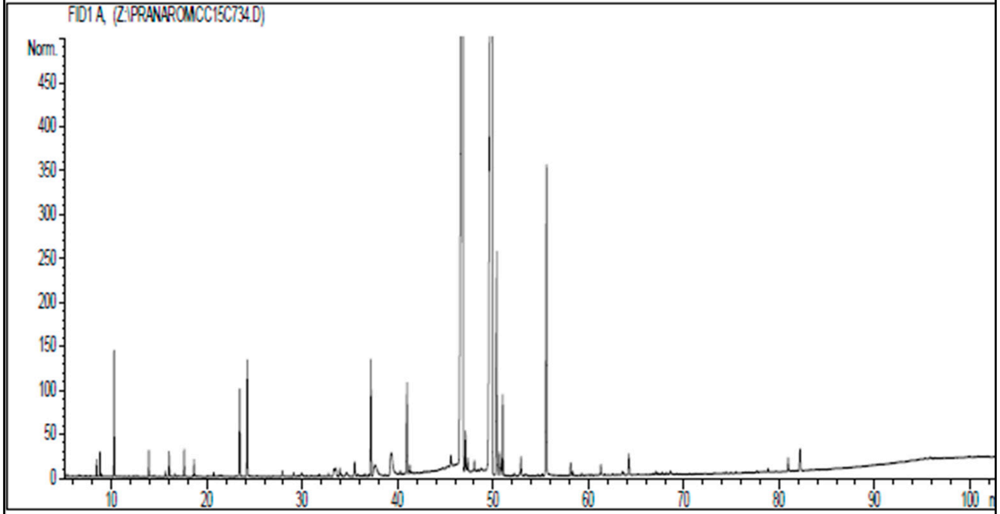

Figure S7 (page 2/5) – Certificate of the chemical analysis for the lot number OF20407 of *Cymbopogon citratus* (DC.) Staph. (lemongrass) provided by Pranarôm. Screen capture made on 1st September 2020.

Tableau de résultats 1 : CYMBOPOGON CITRATUS

LOT N° OF20407

Pics	Temps de rétention	Constituants	%
1	4,7	ACETONE	0,02
2	5,4	ACETATE D'ETHYLE	0,01
3	6,1	ISOVALERALDEHYDE	0,01
4	6,2	ETHANOL	0,01
5	7,7	3,3,5-TRIMETHYL-1,4-HEXADIENE	0,01
6	8,4	TRICYCLENE	0,14
7	8,8	α-PINENE	0,22
8	8,9	α-THUYENE	0,03
9	9,1	2,6-DIMETHYL-2,4-HEPTADIENE	0,01
10	10,5	CAMPHENE	1,17
11	11,0	HEXANAL	0,01
12	12,3	β-PINENE	0,01
13	12,8	SABINENE	0,01
14	13,0	4-HEPTANONE	0,02
15	14,3	Δ3-CARENE	0,01
16	14,8	β-MYRCENE	0,10
17	15,1	α-PHELLANDRENE	0,01
18	15,9	α-TERPINENE + HEPTANAL	0,01
19	16,6	2,3-DEHYDRO-1,8-CINEOLE	0,04
20	16,9	ALCOOL ISOAMYLIQUE	0,04
21	17,1	LIMONENE	1,84
22	16,6	β-PHELLANDRENE	0,03
23	17,7	1,8-CINEOLE	0,01
24	17,9	MENTHATRIENE ISOMERE + 2-HEXENAL	0,01
25	18,9	Cis-β-OCIMENE	0,30
26	19,8	γ-TERPINENE	0,01
27	20,0	Trans-β-OCIMENE	0,19
28	21,2	p-CYMENE	0,01
34	25,5	6-METHYL-5-HEPTEN-2-ONE	1,05
35	25,6	Trans-3-HEXEN-1-OL	0,03
36	26,1	1-HEXANOL	0,01
37	26,7	Cis-OXYDE DE ROSE	0,01
38	26,7	NONANAL	0,02
39	27,9	ALLO-OCIMENE	0,01
40	28,1	Cis-3-HEXEN-1-OL	0,01
41	29,4	TRIMETHYL ANISALDEHYDE	0,11
42	30,6	PERILLENE	0,06
43	30,8	ALDEHYDE FURANIQUE	0,01
44	31,1	ACETATE DE 7-METHYL-4-OCTYLE	0,03
45	31,3	Epi-PHOTOCITRAL ISOMERE	0,13
46	31,9	ALDEHYDE TERPENIQUE	0,02

Figure S7 (page 3/5) – Certificate of the chemical analysis for the lot number OF20407 of *Cymbopogon citratus* (DC.) Staph. (lemongrass) provided by Pranarôm. Screen capture made on 1st September 2020.

Tableau de résultats 2 : CYMBOPOGON CITRATUS

LOT N° OF20407

Pics	Temps de rétention	Constituants	%
47	32,4	Cis-OXYDE DE LINALOL	0,02
48	33,3	6-METHYL-5-HEPTEN-2-OL	0,02
49	33,9	α-CUBEBENE	0,02
50	34,0	Trans-THUYANOL	0,01
51	34,3	Trans-OXYDE DE LINALOL	0,02
52	34,6	EPOXY-4,8-TERPINOLENE	0,03
53	35,0	CITRONELLAL	0,22
54	35,4	ISOGERANIAL	0,13
55	35,6	YLANGENE + CYCLOSATIVENE	0,19
56	36,0	α-CAMPHOLENE ALDEHYDE	0,01
57	36,3	α-COPAENE	0,02
58	36,5	DECANAL	0,16
59	37,0	ISONERAL	0,28
60	37,5	BENZALDEHYDE	0,03
61	37,6	α-BOURBONENE	0,02
62	37,8	β-BOURBONENE	0,02
63	38,0	4-UNDECANONE	0,04
64	38,6	SESQUITERPENE	0,07
65	38,9	LINALOL	1,05
66	39,2	PHOTOCITRAL A	0,82
67	39,8	SESQUITERPENE	0,06
68	40,2	Trans-p-MENTH-2-EN-1-OL	0,05
69	40,8	PHOTOCITRAL B	1,17
70	41,5	ε-CADINENE	0,23
71	42,0	SESQUITERPENE	0,04
72	42,4	β-ELEMENE	0,15
73	42,6	COMPOSÉ Mw=166	0,16
74	43,0	β-CARYOPHYLLENE + TERPINENE-4-OL	1,42
75	43,5	6,9-GUAIADIENE	0,03
76	44,1	BENZOATE DE METHYLE	0,02
77	44,2	SESQUITERPENE	0,02
78	46,5	ACETATE DE CITRONELLYLE	0,06
79	46,7	E-β-FARNESENE	0,03
80	47,0	SESQUITERPENE	0,03
86	49,4	FORMIATE DE GERANYLE	0,12
87	50,1	GERMACRENE D	0,16
88	50,6	β-BISABOLENE	0,08
89	51,5	**GERANIAL**	**40,15**
90	51,7	ISOPIPERITENOL	0,02
91	52,1	**ACETATE DE GERANYLE**	3,33
92	52,3	CITRONELLOL	0,11

Figure S7 (page 4/5) – Certificate of the chemical analysis for the lot number OF20407 of *Cymbopogon citratus* (DC.) Staph. (lemongrass) provided by Pranarôm. Screen capture made on 1st September 2020.

Tableau de résultats 3 : CYMBOPOGON CITRATUS

LOT N° OF20407

Pics	Temps de rétention	Constituants	%
93	52,5	δ-CADINENE	0,34
94	52,9	γ-CADINENE	1,11
95	53,2	CUMINAL	0,01
96	53,4	CADINA-1,4-DIENE	0,04
97	54,2	NEROL	0,21
98	54,7	Cis-ISOGERANIOL	0,01
99	54,8	α-AMORPHENE	0,05
100	55,1	Trans-ISOGERANIOL	0,01
101	56,4	CUPARENE	0,03
102	57,0	**GERANIOL**	**6,07**
103	57,2	CALAMENENE	0,03
104	58,0	COMPOSÉ FURANIQUE	0,01
105	60,0	BUTYRATE DE GERANYLE	0,14
106	60,1	EPI-CUBEBOL	0,06
107	62,0	α-CALACORENE + PHYTADIENE ISOMERE	0,03
108	62,3	OXYDE D'HUMULENE	0,01
109	62,9	CUBEBOL	0,17
110	63,2	CYCLODODECENE	0,01
111	64,1	SESQUITERPENOL	0,01
112	64,5	CETONE CYCLOTERPENIQUE	0,09
113	65,2	OXYDE D'ISOCARYOPHYLLENE	0,06
114	65,7	OXYDE DE CARYOPHYLLENE	0,33
115	66,0	EPOXYDE SESQUITERPENIQUE	0,01
116	66,8	EPOXY-6,7-HUMULENE	0,01
117	67,6	ACIDE CAPRYLIQUE	0,04
118	68,1	CETONE TERPENIQUE	0,03
119	68,7	SESQUITERPENOL	0,03
120	68,8	CARYOPHYLLENOL	0,02
121	69,1	Epi-CUBENOL	0,03
122	69,6	CUBENOL	0,02
123	69,9	ELEMOL	0,04
124	70,4	ESTER GERANIQUE	0,01
125	72,3	COMPOSE ALIPHATIQUE	0,01
126	73,6	TRIMETHYL PENTADECANONE	0,02
127	75,0	SESQUITERPENOL	0,01
128	76,7	SESQUITERPENOL	0,02
129	78,0	ACETATE D'EUGENYLE	0,01
130	78,6	ACIDE CAPRIQUE	0,02
131	79,5	ACIDE NERIQUE	0,06
132	81,6	ACIDE GERANIQUE	0,21
133	82,3	ISOEUGENOL	0,35
134	84,2	EPOXYDE SESQUITERPENIQUE Mw=220	0,01
135	96,9	COMPOSÉ AROMATIQUE Mw=286	0,02
136	101,2	COMPOSÉ ALIPHATIQUE	0,02
137	102,0	DIONE ALIPHATIQUE	0,01
		**TOTAL**	**99,91**

Date de l'analyse – date of the analysis : Mai 2015,

Pranarôm Int.

Figure S7 (page 5/5) – Certificate of the chemical analysis for the lot number OF20407 of *Cymbopogon citratus* (DC.) Staph. (lemongrass) provided by Pranarôm. Screen capture made on 1st September 2020.

Figure S8 (page 1/7) – Certificate of the chemical analysis for the lot number OF19205 of *Lavandula stoechas* L. (Spanish lavender) provided by Pranarôm. Screen capture made on 1st September 2020.

PRANARŌM
international

FICHE D'ANALYSE – ANALYSIS SHEET

Huile essentielle – Essential oil

Nom botanique – botanical name :	***Lavandula stoechias* Organic**
Nom commun – french name :	LAVANDE STOECHADE BIOLOGIQUE
Numéro du lot – lot number:	**OF19205**
Origine - origin: ----	PRANAROM - FRANCE
Partie de la plante – part of the plant :	SOMMITE FLEURIE
Date de distillation – distillation date :	06/2015
Date de peremption – out of date :	06/2020

Caractéristiques d'analyse - analysis characteristics:

CPG - SM HEWLETT PACKARD / CPG-FID
Colonne : HP INNOWAX 60-0.5-0.25
Programmation de température : 6 mn à 50 °C – 2 °C/mn→150°C
Gaz vecteur He : 23 psis 4 °C/mn→250°C - 20mn à 250 °C

Caractéristiques physiques – physical characteristics:

Aspect - physical state	Liquide limpide
Couleur - colour	Jaune clair
Odeur - odour	Caractéristique, camphrée
Densité à 20°C - density	0,944
Densité à 15°C - density	0,947
Indice de réfraction à 20°C - refractive index	1,472 4
Pouvoir rotatoire à 20°C - optical rotation	+ 21,5 °
Miscibilité à l'éthanol à 75% - miscibility	1,3 volumes d'alcool / 1 volume d'HE
Point d'éclair - flashpoint	60,1 °C

Analyses pesticides – pesticide analysis :

**Pesticides Organochlorés : Dosage par GC MS détecteur XSD (méthode multirésidus interne validée selon la norme NF V03-110)**<br>**Liste des pesticides recherchés (Pharmacopée Européenne)**: Alachlor, Aldrine, Bromophos Ethyl, Bromophos Methyl, Chlordane, Chlorfenvinphos, Chlorpyriphos, Chlorpyriphos Methyl, Chlorthal Dimethyl, Cyfluthrine, Cyhalothrine lambda, Cypermethrine, Dichlofluanide, Dichlorvos, Dicofol (Kelthane), Dieldrine, Endosulfan, Endosulfan sulfate, Endrine, Fenchlorphos (Ronnel), Fenchlorphos-oxon, Fenvalerate, Fluvalinate, Heptachlor, Heptachlor epoxide, Hexachlorobenzene, Hexachlorocyclohexane α, Hexachlorocyclohexane β, Hexachlorocyclohexane δ, Hexachlorocyclohexane ε, Lindane, Methoxychlore, Mirex, Naled, o,p'-DDD , o,p'-DDE, o,p'-DDT, Oxychlordane, p,p'-DDD, p,p'-DDE, p,p'-DDT, Pentachloroaniline, Pentachloroanisole, Permethrine , Phosalone, Procymidone, Profenophos, Prothiofos, Quintozene, S421, Tecnazene, Tetradifon, Vinclozoline	**Résultats**<br>< LMR*<br>* Limite Maximale de Résidus autorisée
**Pesticides Organophosphorés : Dosage par GC MS détecteur FPD (méthode multirésidus interne validée selon la norme NF V03-110)**<br>**Liste des pesticides recherchés (Pharmacopée Européenne)**: Acephate, Azinphos Ethyl, Azinphos Méthyl, Bromophos Ethyl, Bromophos Méthyl, Chlorfenvinphos, Chlorpyriphos, Chlorpyriphos Methyl, Diazinon, Dichlorvos, Dimethoate, Ethion, Etrimphos, Fenchlorphos (Ronnel), Fenchlorphos-oxon, Fenitrothion, Fensulfothion (Dasanit), Fensulfothion-oxon, Fensulfothion-oxon-sulfone, Fensulfothion-sulfone, Fenthion, Fenthion-oxon, Fenthion-oxon-sulfone, Fenthion-oxon-sulfoxyde, Fenthion-sulfone, Fenthion-sulfoxyde, Fonofos, Malaoxon, Malathion, Mecarbam, Methacrifos, Methamidophos (Monitor), Methidathion, Monocrotophos, Naled, Omethoate, Paraoxon, Paraoxon Méthyl, Parathion Ethyl, Parathion Methyl, Phosalone, Phosmet, Pirimiphos Ethyl, Pirimiphos Méthyl, Profenophos, Prothiofos, Quinalphos.	**Résultats**<br>< LMR*<br>* Limite Maximale de Résidus autorisée

Profil CHROMATOGRAPHIQUE

Figure S8 (page 2/7) – Certificate of the chemical analysis for the lot number OF19205 of *Lavandula stoechas* L. (Spanish lavender) provided by Pranarôm. Screen capture made on 1st September 2020.

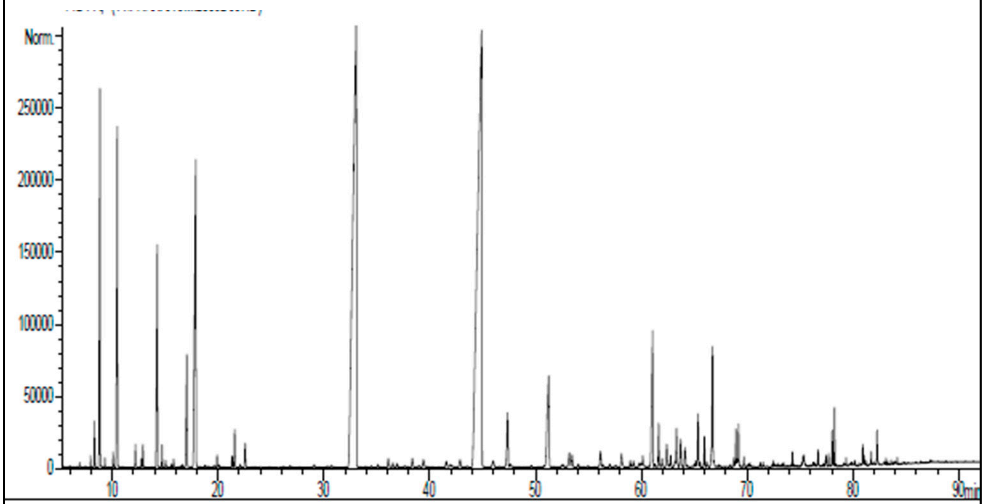

Tableau de résultats 1 : LAVANDULA STOECHAS

OF19205

Pics	Temps de rétention	Constituants	%
1	4,6	ACETONE	0,02
2	5,0	METHYL PROPYL CYCLOPENTANE	0,01
3	5,9	2-METHYLBUTENAL	0,01
4	6,0	ISOVALERALDEHYDE	0,01
5	6,6	2-ETHYL FURANE	0,01
6	6,9	METHYL PROPENYL CYCLOPENTANE	0,03
7	7,1	TERPENE Mw=124	0,01
[illegible]	[illegible]	[illegible]	[illegible]
[illegible]	[illegible]	[illegible]	[illegible]
13	9,0	PRENOL	0,02
14	9,3	TOLUENE	0,08
15	10,1	α-FENCHENE	0,15
16	10,5	**CAMPHENE**	**3,94**
17	10,9	HEXANAL	0,01
18	12,1	β-PINENE	0,23
19	12,7	SABINENE	0,08
20	12,9	PINADIENE	0,23
21	13,1	THUYADIENE	0,01
22	14,3	**Δ3-CARENE**	**2,89**
23	14,7	β-MYRCENE	0,21
24	15,0	α-PHELLANDRENE	0,08
25	15,2	ψ-LIMONENE	0,01
26	15,6	o-CYMENE	0,04
27	15,8	α-TERPINENE	0,08
28	16,5	2,3-DEHYDRO-1,8-CINEOLE	0,02
29	16,6	MENTHATRIENE ISOMERE	0,03
30	16,8	ISOSYLVESTRENE	0,01
31	17,5	LIMONENE	1,65
32	17,8	**1,8-CINEOLE**	**6,57**
33	18,0	MENTHATRIENE ISOMERE	0,08
34	18,2	2-HEXENAL	0,02
35	18,7	2-PENTYL FURANE	0,03
36	19,0	Cis-β-OCIMENE	0,02
37	19,1	2-METHYL-1-HEPTEN-6-ONE	0,01
38	19,6	TERPENE ISOMERE	0,04
39	19,9	γ-TERPINENE	0,15
40	20,1	Trans-β-OCIMENE	0,03
41	20,6	MENTHATRIENE ISOMERE	0,01
42	21,3	m-CYMENE	0,14
43	21,5	p-CYMENE	0,47
44	22,0	ISOTERPINOLENE	0,05
45	22,5	TERPINOLENE	0,33

Figure S8 (page 3/7) – Certificate of the chemical analysis for the lot number OF19205 of *Lavandula stoechas* L. (Spanish lavender) provided by Pranarôm. Screen capture made on 1st September 2020.

Tableau de résultats 2 : LAVANDULA STOECHAS

OF19205

Pics	Temps de rétention	Constituants	%
46	23,2	DIMETHYLSTYRENE ISOMERE	0,01
47	24,0	CYMENE ISOMERE	0,01
48	24,9	ACETATE DE 3-HEXEN-1-OL	0,01
49	25,9	PINOL	0,01
50	26,2	ACETATE DE cis-3-HEXENYLE	0,02
51	26,8	6-METHYL-5-HEPTEN-2-ONE	0,03
52	29,1	COMPOSÉ AROMATIQUE	0,06
53	30,3	3-HEXEN-1-OL	0,03
54	30,7	ACETATE D'OCT-1-EN-3-YLE	0,04
55	31,0	MYRTENYL METHYL ETHER	0,01
56	33,0	**FENCHONE**	**25,23**
57	33,9	PERILLENE	0,02
58	34,4	2-METHYLBUTYRATE D'HEXYLE	0,03
59	35,1	DIMETHYLSTYRENE ISOMERE	0,07
60	36,0	α,p-DIMETHYLSTYRENE	0,18
61	36,5	CIS-OXYDE DE LINALOL	0,08
62	36,9	1-OCTEN-3-OL	0,08
63	37,6	Cis-1,2-EPOXYDE DE LIMONENE	0,03
64	37,9	COMPOSÉ AROMATIQUE Mw=150	0,02
65	38,3	CAMPHENILONE	0,17
[illegible]	[illegible]	[illegible]	[illegible]
70	39,7	Trans-OXYDE DE LINALOL	0,02
71	41,5	CYCLOSATIVENE + YLANGENE	0,14
72	42,0	α-CAMPHOLENE ALDEHYDE	0,15
73	42,9	α-COPAENE	0,18
74	43,5	CHRYSANTHENONE	0,06
75	44,9	**CAMPHRE**	**29,60**
76	45,5	SESQUITERPENE	0,01
77	45,8	α-GURJUNENE	0,03
78	46,0	ALCOOL CAMPHOLENIQUE	0,16
79	46,5	β-CUBEBENE	0,03
80	47,3	LINALOL	1,00
81	47,4	ALCOOL ALIPHATIQUE	0,02
82	47,6	ALCOOL CAMPHOLENIQUE ISOMERE + CETONE TERP,	0,05
83	47,8	1-OCTANOL	0,08
84	48,5	OXYDE TERPENIQUE	0,01
85	48,8	Trans-p-MENTH-2-EN-1-OL	0,02
86	49,2	DIMETHYL SULFOXIDE	0,01
87	49,6	PINOCARVONE	0,04
88	50,4	FORMIATE DE BORNYLE	0,02
89	50,8	NOPINONE	0,03
90	51,2	**ACETATE DE BORNYLE**	**2,60**

Figure S8 (page 4/7) – Certificate of the chemical analysis for the lot number OF19205 of *Lavandula stoechas* L. (Spanish lavender) provided by Pranarôm. Screen capture made on 1st September 2020.

Tableau de résultats 3 : LAVANDULA STOECHAS

OF19205

Pics	Temps de rétention	Constituants	%
91	51,3	FENCHOL	0,20
92	51,7	SESQUITERPENE Mw=202	0,02
93	52,5	β-ELEMENE + CETONE TERPENIQUE	0,05
94	52,6	HYDRATE DE CAMPHENE + β-CUBEBENE	0,05
95	53,2	β-CARYOPHYLLENE	0,34
96	53,4	TERPINENE-4-OL	0,25
97	54,0	HOTRIENOL	0,06
98	55,0	DEHYDROSABINACETONE	0,03
99	55,9	Trans-p-2,8-MENTHADIEN-1-OL	0,01
100	56,1	MYRTENAL	0,33
101	56,4	CAMPHELINOL	0,05
102	56,5	SABINACETONE	0,02
103	56,9	CADINA-3,5-DIENE	0,06
104	57,0	ALCOOL TERPENIQUE	0,01
105	57,6	SESQUITERPENE	0,05
106	58,0	ALLO-AROMADENDRENE	0,15
107	58,1	Cis-VERBENOL + Trans-PINOCARVEOL	0,13
108	58,9	MENTHADIENOL ISOMERE	0,09
109	59,2	ZONARENE	0,09
110	59,3	LACTONE LAVANDE	0,02
111	59,5	Cis-p-2,8-MENTHADIEN-1-OL	0,02
112	59,8	δ-TERPINEOL	0,06
113	59,9	α-HUMULENE	0,07
114	60,1	Trans-VERBENOL	0,19
115	60,3	γ-SELINENE	0,02
116	60,6	γ-CURCUMENE	0,02
117	61,0	**ACETATE DE MYRTENYLE**	**2,51**
118	61,2	HEPTADECANE	0,06
119	61,4	α-TERPINEOL	0,25
120	61,6	BORNEOL	0,32
121	62,0	LEDENE	0,15
122	62,4	VERBENONE	0,22
123	62,5	SESQUITERPENE	0,20
124	62,6	GERMACRENE D	0,05

Figure S8 (page 5/7) – Certificate of the chemical analysis for the lot number OF19205 of *Lavandula stoechas* L. (Spanish lavender) provided by Pranarôm. Screen capture made on 1st September 2020.

129	63,7	α-SELINENE + SESQUITERPENE	0,45
130	64,1	BICYCLOGERMACRENE + CARVONE	0,32
131	65,0	SESQUITERPENE Mw=202	0,04
132	65,1	ACETATE DE GERANYLE	0,08
133	65,4	δ-CADINENE	0,73
134	65,6	γ-CADINENE	0,06
135	66,0	δ-SELINENE	0,39

Tableau de résultats 4 : LAVANDULA STOECHAS

OF19205

Pics	Temps de rétention	Constituants	%
136	66,2	p-METHYLACETOPHENONE	0,10
137	66,7	SELINA-3,7-DIENE	1,52
138	66,8	MYRTENOL	0,40
139	67,3	α-AMORPHENE	0,03
140	67,5	SESQUITERPENE Mw=202	0,02
141	68,4	SESQUITERPENE Mw=204	0,05
142	68,7	Trans-CARVEOL	0,10
143	68,9	m-CYMEMOL + CALAMENENE	0,38
144	69,0	GERANIOL	0,10
145	69,2	p-CYMENE-8-OL	0,43
146	69,4	ESTER ALIPHATIQUE	0,02
147	69,6	ESTER MYRTENIQUE	0,13
148	70,1	COMPOSÉ Mw=220	0,04
149	70,3	ESTER MYRTENIQUE	0,05
150	71,2	COMPOSÉ INDENIQUE Mw=188	0,05
151	71,5	Epi-CUBEBOL	0,07
152	72,5	α-CALACORENE	0,06
153	72,6	PIPERITONE	0,03
154	72,7	OXYDE D'HUMULENE	0,03
155	72,9	PALUSTROL	0,04
156	73,2	CUBEBOL	0,04
157	73,4	COMPOSÉ AROMATIQUE	0,05
158	74,2	COMPOSÉ Mw=218	0,14
159	74,5	COMPOSÉ AROMATIQUE	0,03
160	74,8	OXYDE D'ISOCARYOPHYLLENE	0,02
161	75,2	OXYDE DE CARYOPHYLLENE	0,12
162	75,4	METHYL EUGENOL	0,16
163	75,6	CETONE TERPENIQUE	0,04
164	75,5	SESQUITERPENOL	0,02
165	76,2	SESQUITERPENOL	0,06
166	76,4	COMPOSÉ CETONIQUE	0,05
167	76,7	LEDOL	0,22
168	77,2	EPOXY-6,7-HUMULENE	0,03
169	77,3	GLEENOL	0,06
170	77,5	Epi-CUBENOL	0,10
171	77,6	CUBENOL	0,14
172	77,8	SESQUITERPENOL	0,15
173	78,0	COPAENOL ISOMERE	0,24
174	78,2	VIRIDIFLOROL	0,52
175	78,4	ROSIFOLIOL	0,02
176	79,1	TRIMETHYL PENTADECANONE	0,04
177	79,3	SPATHULENOL	0,10
178	79,7	SESQUITERPENOL	0,07
179	79,9	SESQUITERPENOL	0,08
180	80,2	SESQUITERPENOL Mw=220	0,07

Figure S8 (page 6/7) – Certificate of the chemical analysis for the lot number OF19205 of *Lavandula stoechas* L. (Spanish lavender) provided by Pranarôm. Screen capture made on 1st September 2020.

Tableau de résultats 5 : LAVANDULA STOECHAS

OF19205

Pics	Temps de rétention	Constituants	%
181	80,6	T-CADINOL	0,04
182	80,9	DEHYDROVIRIDIFLOROL	0,22
183	81,0	α-MUUROLOL	0,10
184	81,3	SESQUITERPENOL	0,06
185	81,7	GERANYL-α-TERPINENE	0,16
186	81,9	SPATHULENOL ISOMERE	0,03
187	82,3	α-CADINOL	0,36
188	82,4	CADALENE	0,02
189	82,9	SESQUITERPENOL	0,07
190	83,1	SESQUITERPENONE Mw=218	0,06
191	83,6	GERANYL-p-CYMENE ISOMERE	0,04
192	83,8	SESQUITERPENOL	0,06
193	84,1	MAALIOL ISOMERE	0,07
194	84,2	ACETATE D'EUGENYLE	0,01
195	84,8	COMPOSÉ METHOXY AROMATIQUE	0,02
196	86,4	COMPOSÉ AROMATIQUE	0,02
197	87,3	SESQUITERPENONE Mw=218	0,02
		**TOTAL**	**99,69**

Date de l'analyse – date of the analysis : Juin 2015,

Pranarôm Int.
C. Schulze
Contrôle qualité

Figure S8 (page 7/7) – Certificate of the chemical analysis for the lot number OF19205 of *Lavandula stoechas* L. (Spanish lavender) provided by Pranarôm. Screen capture made on 1st September 2020.

Figure S9 (page 1/5) – Certificate of the chemical analysis for the lot number OF22429 of *Origanum compactum* Benth. (oregano) provided by Pranarôm. Screen capture made on 1st September 2020.

[illegible]

Huile essentielle – Essential oil

Nom botanique – botanical name :	***Origanum compactum***
Nom commun – french name :	ORIGAN COMPACT
Numéro du lot – lot number :	**OF22429**
Origine - origin : ---	PRANARÔM - MAROC
Partie de la plante – part of the plant :	SOMMITÉ FLEURIE
Date de distillation – distillation date :	06/2015
Date de péremption – out of date :	02/2021

Caractéristiques d'analyse – analysis characteristics:

CPG - SM HEWLETT PACKARD / CPG-FID
Colonne : HP INNOWAX 60-0.5-0.25
Programmation de température : 6 mn à 50°C -2°C/mn→250°C-10mn à 250°C
Gaz vecteur He : 22 psis

Caractéristiques physiques – physical characteristics:

Aspect – physical state	Liquide limpide
Couleur - colour	Jaune d'or clair
Odeur - odour	Caractéristique, phénolique
Densité à 20°C - density	0,938
Densité à 15°C - density	0,941
Indice de réfraction à 20°C - refractive index	1,505 2
Pouvoir rotatoire à 20°C – optical rotation	0 °
Miscibilité à l'éthanol à 80% - miscibility	1,1 volumes d'alcool / 1 volume d'HE
Point d'éclair : SETAFLASH - flashpoint	63,4 °C

Analyses pesticides – pesticide analysis :

**Pesticides Organochlorés : Dosage par GC MS détecteur XSD (méthode multirésidus interne validée selon la norme NF V03-110)** **Liste des pesticides recherchés (Pharmacopée Européenne)**: Alachlor, Aldrine, Bromophos Ethyl, Bromophos Methyl, Chlordane, Chlorfenvinphos, Chlorpyriphos, Chlorpyriphos Methyl, Chlorthal Dimethyl, Cyfluthrine, Cyhalothrine lambda, Cypermethrine, Dichlofluanide, Dichlorvos, Dicofol (Kelthane), Dieldrine, Endosulfan, Endosulfan sulfate, Endrine, Fenchlorphos (Ronnel), Fenchlorphos-oxon, Fenvalerate, Fluvalinate, Heptachlor, Heptachlor epoxide, Hexachlorobenzene, Hexachlorocyclohexane α, Hexachlorocyclohexane β, Hexachlorocyclohexane δ, Hexachlorocyclohexane ε, Lindane, Methoxychlore, Mirex, Naled, o,p'-DDD , o,p'-DDE, o,p'-DDT, Oxychlordane, p,p'-DDD, p,p'-DDE, p,p'-DDT, Pentachloroaniline, Pentachloroanisole, Permethrine , Phosalone, Procymidone, Profenophos, Prothiofos, Quintozene, S421, Tecnazene, Tetradifon, Vinclozoline	**Résultats** < LMR* * Limite Maximale de Résidus autorisée
**Pesticides Organophosphorés : Dosage par GC MS détecteur FPD (méthode multirésidus interne validée selon la norme NF V03-110)** **Liste des pesticides recherchés (Pharmacopée Européenne)**: Acephate, Azinphos Ethyl, Azinphos Méthyl, Bromophos Ethyl, Bromophos Méthyl, Chlorfenvinphos, Chlorpyriphos, Chlorpyriphos Methyl, Diazinon, Dichlorvos, Dimethoate, Ethion, Etrimphos, Fenchlorphos (Ronnel), Fenchlorphos-oxon, Fenitrothion, Fensulfothion (Dasanit), Fensulfothion-oxon, Fensulfothion-oxon-sulfone, Fensulfothion-sulfone, Fenthion, Fenthion-oxon, Fenthion-oxon-sulfone, Fenthion-oxon-sulfoxyde, Fenthion-sulfone, Fenthion-sulfoxyde, Fonofos, Malaoxon, Malathion, Mecarbam, Methacrifos, Methamidophos (Monitor), Methidathion, Monocrotophos, Naled, Omethoate, Paraoxon, Paraoxon Méthyl, Parathion Ethyl, Parathion Methyl, Phosalone, Phosmet, Pirimiphos Ethyl, Pirimiphos Méthyl, Profenophos, Prothiofos, Quinalphos.	**Résultats** < LMR* * Limite Maximale de Résidus autorisée

Profil CHROMATOGRAPHIQUE

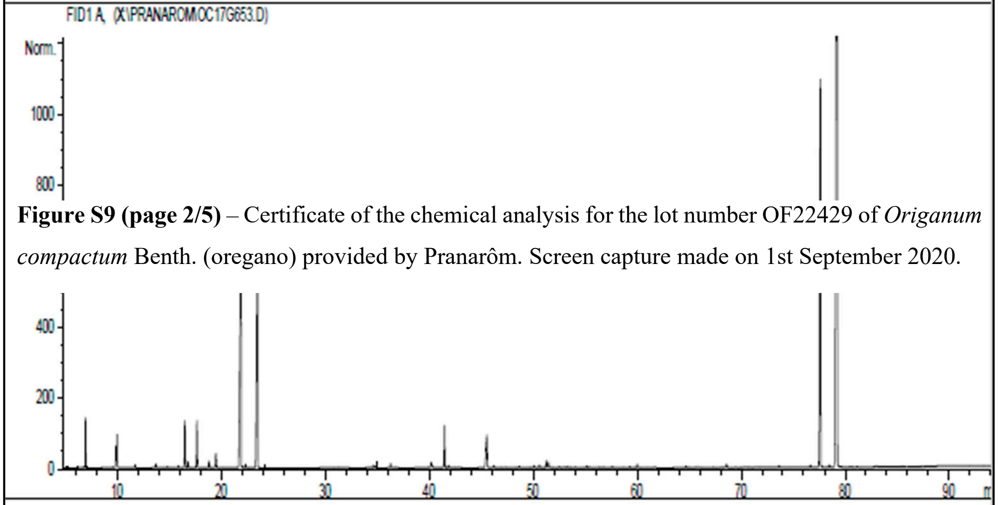

Figure S9 (page 2/5) – Certificate of the chemical analysis for the lot number OF22429 of *Origanum compactum* Benth. (oregano) provided by Pranarôm. Screen capture made on 1st September 2020.

Tableau de résultats 1 : ORIGANUM COMPACTUM

OF22429

Pics	Temps de rétention	Constituants	%
1	5,1	ACETONE	0,02
2	6,2	FORMIATE D'ETHYLE	0,02
3	6,8	2-METHYL BUTANAL	0,04
4	6,9	ISOVALERALDEHYDE	0,84
5	7,5	2-ETHYL FURANE	0,01
6	9,4	TRICYCLENE	0,01
7	9,6	ISOVALERATE DE METHYLE	0,02
8	9,8	α-PINENE	0,59
9	9,9	α-THUYENE	0,88
10	11,6	PENTANOATE D'ETHYLE	0,01
11	11,7	CAMPHENE	0,09
12	13,7	β-PINENE	0,12
13	14,5	SABINENE	0,01
14	14,8	PINADIENE	0,01
15	15,0	4-METHYL-3-PENTEN-2-ONE	0,03
16	15,8	Δ3-CARENE	0,07
17	16,0	3-HEPTANONE	0,01
18	16,5	β-MYRCENE	1,47
19	16,8	α-PHELLANDRENE	0,19
20	17,0	ψ-LIMONENE	0,01
21	17,6	α-TERPINENE	1,57
26	20,1	2-HEXENAL	0,01
27	20,9	Cis-β-OCIMENE	0,01
**28**	**21,8**	**γ-TERPINENE**	**12,79**
29	22,0	Trans-β-OCIMENE	0,07
30	22,3	3-OCTANONE	0,11
31	23,1	m-CYMENE	0,01
**32**	**23,4**	**p-CYMENE**	**10,22**
33	24,2	TERPINOLENE	0,08
34	30,6	3-HEXEN-1-OL	0,01
35	31,2	3-OCTANOL	0,01
36	31,7	MENTHATRIENE ISOMERE	0,01
37	33,2	α-THUYONE	0,01
38	33,9	CETONE TERPENIQUE	0,01
39	34,2	MENTHATRIENE ISOMERE	0,03
40	34,6	α,p-DIMETHYLSTYRENE	0,09
41	34,8	MENTHATRIENE ISOMERE	0,06
42	34,9	1-OCTEN-3-OL	0,20
43	35,2	FURFURAL	0,01
44	36,3	Trans-THUYANOL	0,15
45	36,6	EPOXY-4,8-TERPINOLENE	0,01

Figure S9 (page 3/5) – Certificate of the chemical analysis for the lot number OF22429 of *Origanum compactum* Benth. (oregano) provided by Pranarôm. Screen capture made on 1st September 2020.

Tableau de résultats 2 : ORIGANUM COMPACTUM

OF22429

Pics	Temps de rétention	Constituants	%
46	36,7	Trans-OXYDE DE LINALOL	0,02
47	37,6	ALDEHYDE TERPENIQUE	0,01
48	38,6	α-COPAENE	0,01
49	39,6	EPOXYDE TERPENIQUE	0,01
50	39,9	EPOXYDE TERPENIQUE	0,03
51	40,1	CAMPHRE	0,23
52	40,2	CÉTONE TERPENIQUE Mw=152	0,07
53	41,0	β-BOURBONENE	0,01
54	41,4	α-GURJUNENE	0,01
55	41,5	LINALOL	1,36
56	41,6	1-NONEN-3-OL	0,02
57	41,8	Cis-THUYANOL	0,07
58	42,1	β1-CUBEBENE	0,02
59	42,5	4-ACETYL-1-METHYLCYCLOHEXENE	0,02
60	42,8	Trans-p-MENTH-2-EN-1-OL	0,03
61	43,5	MENTHADIENE ISOMERE	0,02
62	45,4	METHYL CARVACROL ETHER	0,73
63	45,5	β-CARYOPHYLLENE + TERPINENE-4-OL	1,36
64	46,2	Cis-DIHYDROCARVONE + AROMADENDRENE	0,10
65	46,9	Trans-DIHYDROCARVONE	0,03
66	47,3	Cis-p-MENTH-2-EN-1-OL	0,02
67	48,4	α-HIMACHALENE	0,02
68	48,6	ALLO-AROMADENDRENE + COMPOSÉ Mw=152	0,05
69	49,3	ZONARENE	0,01
70	49,7	δ-TERPINEOL	0,02
71	50,0	α-HUMULENE	0,09
72	50,6	NERAL	0,08
73	50,8	CARVOTANACETONE	0,02
74	51,2	α-TERPINEOL	0,26
75	51,4	BORNEOL	0,20
76	51,6	SESQUITERPENE	0,01
77	51,8	LEDENE	0,02
78	52,4	VERBENONE + β-HIMACHALENE	0,06
79	52,9	COMPOSÉ TERPENIQUE Mw=152	0,04
80	53,2	β-BISABOLENE	0,05

Figure S9 (page 4/5) – Certificate of the chemical analysis for the lot number OF22429 of *Origanum compactum* Benth. (oregano) provided by Pranarôm. Screen capture made on 1st September 2020.

85	54,8	1-DECANOL	0,01
86	55,1	δ-CADINENE	0,08
87	55,4	γ-CADINENE	0,04
88	56,3	METHYLACETOPHENONE	0,01
89	56,4	MENTHADIENOL ISOMERE	0,01
90	56,7	CUMINAL	0,01

Tableau de résultats 3 : ORIGANUM COMPACTUM

OF22429

Pics	Temps de rétention	Constituants	%
91	56,8	MYRTENOL + CURCUMENE ISOMERE	0,01
92	57,6	SABINOL ISOMERE	0,04
93	57,7	2-HYDROXY PIPERITONE	0,01
94	58,1	SABINOL ISOMERE	0,01
95	58,6	CURCUMENE ISOMERE	0,01
96	59,3	Trans-CARVEOL	0,04
97	59,7	CALAMENENE	0,01
98	60,0	p-CYMENE-8-OL + ACETATE DE CARVACRYLE	0,12
99	60,9	ESTER TERPENIQUE	0,01
100	61,7	ESTER TERPENIQUE	0,03
101	64,7	METHYL CARVACROL	0,04
102	65,6	COMPOSÉ Mw=166	0,02
103	67,3	METHYL PROPENYL PHENOL Mw=148	0,01
104	68,3	OXYDE D'ISOCARYOPHYLLENE	0,01
105	68,6	OXYDE DE CARYOPHYLLENE	0,16
106	73,6	CUMINOL	0,09
107	75,4	SPATHULENOL	0,01
108	76,6	ISOTHYMOL	0,08
109	77,2	COMPOSÉ ACÉTOXY CÉTONIQUE	0,01
110	77,6	**THYMOL**	**14,89**
111	78,4	ISOCARVACROL	0,09
112	79,2	**CARVACROL**	**48,08**
113	80,4	COMPOSÉ PHÉNOLIQUE	0,02
114	81,1	COMPOSÉ PHÉNOLIQUE	0,01
115	83,0	VINYL-QUAJACOL	0,03
116	83,8	CARYOPHYLLA-3,7-DIEN-6-OL	0,01
117	85,5	COMPOSÉ PHÉNOLIQUE	0,01
118	87,4	COMPOSÉ PHÉNOLIQUE	0,02
119	88,9	COMPOSÉ PHÉNOLIQUE	0,03
		**TOTAL**	**99,99**

Date de l'analyse – date of the analysis : Mars 2016,

Pranarôm Int.
C. Schulze
Contrôle qualité

Figure S9 (page 5/5) – Certificate of the chemical analysis for the lot number OF22429 of *Origanum compactum* Benth. (oregano) provided by Pranarôm. Screen capture made on 1st September 2020.

PRANARŌM
international

FICHE D'ANALYSE – ANALYSIS SHEET

Huile essentielle - Essential oil

Nom botanique –botanical name: *Cymbopogon martinii*
Nom commun – french name : PALMAROSA
Numéro du lot – lot number: **OF21237**
Origine - origin : --- PRANARÔM -NEPAL
Partie de la plante – part of the plant : PARTIE AERIENNE
Date de distillation – distillation date : 06/2014
Date de péremption – out of date : 03/2020

<u>Caractéristiques d'analyse – analysis characteristics:</u>

CPG - SM HEWLETT PACKARD / CPG-FID
Colonne : HP INNOWAX 60-0.5-0.25
Programmation de température : 6 mn à 50 °C –2 °C/mn→250 °C-10mn à 250 °C
Gaz vecteur He : 22 psis

<u>Caractéristiques physiques – physical characteristics:</u>

Aspect – physical state	Liquide limpide
Couleur - colour	Jaune pâle
Odeur - odour	Rosée et herbacée
Densité à 20°C - density	0,885
Densité à 15°C - density	0,889
Indice de réfraction à 20°C - refractive index	1,475 4
Pouvoir rotatoire à 20°C - optical rotation	- 0,5 °
Miscibilité à l'éthanol à 70% - miscibility	1,2 volumes d'alcool / 1 volume d'HE
Point d'éclair : SETAFLASH - flashpoint	100,8 °C

<u>Analyses pesticides – pesticide analysis :</u>

Pesticides Organochlorés : **Dosage par GC MS détecteur XSD (méthode multirésidus interne validée selon la norme NF V03-110)** **Liste des pesticides recherchés (Pharmacopée Européenne)**: Alachlor, Aldrine, Bromophos Ethyl, Bromophos Methyl, Chlordane, Chlorfenvinphos, Chlorpyriphos, Chlorpyriphos Methyl, Chlorthal Dimethyl, Cyfluthrine, Cyhalothrine lambda, Cypermethrine, Dichlofluanide, Dichlorvos, Dicofol (Kelthane), Dieldrine, Endosulfan, Endosulfan sulfate, Endrine, Fenchlorphos (Ronnel), Fenchlorphos-oxon, Fenvalerate, Fluvalinate, Heptachlor, Heptachlor epoxide, Hexachlorobenzene, Hexachlorocyclohexane α, Hexachlorocyclohexane β, Hexachlorocyclohexane δ, Hexachlorocyclohexane ε, Lindane, Methoxychlore, Mirex, Naled, o,p'-DDD , o,p'-DDE, o,p'-DDT, Oxychlordane, p,p'-DDD, p,p'-DDE, p,p'-DDT, Pentachloroaniline, Pentachloroanisole, Permethrine , Phosalone, Procymidone, Profenophos, Prothiofos, Quintozene, S421, Tecnazene, Tetradifon, Vinclozoline	<u>Résultats</u> < LMR* * Limite Maximale de Résidus autorisée
Pesticides Organophosphorés : **Dosage par GC MS détecteur FPD (méthode multirésidus interne validée selon la norme NF V03-110)** **Liste des pesticides recherchés (Pharmacopée Européenne)**: Acephate, Azinphos Ethyl, Azinphos Méthyl, Bromophos Ethyl, Bromophos Méthyl, Chlorfenvinphos, Chlorpyriphos, Chlorpyriphos Methyl, Diazinon, Dichlorvos, Dimethoate, Ethion, Etrimphos, Fenchlorphos (Ronnel), Fenchlorphos-oxon, Fenitrothion, Fensulfothion (Dasanit), Fensulfothion-oxon, Fensulfothion-oxon-sulfone, Fensulfothion-sulfone, Fenthion, Fenthion-oxon, Fenthion-oxon-sulfone, Fenthion-oxon-sulfoxyde, Fenthion-sulfone, Fenthion-sulfoxyde, Fonofos, Malaoxon, Malathion, Mecarbam, Methacrifos, Methamidophos (Monitor), Methidathion, Monocrotophos, Naled, Omethoate, Paraoxon, Paraoxon Méthyl, Parathion Ethyl, Parathion Methyl, Phosalone, Phosmet, Pirimiphos Ethyl, Pirimiphos Méthyl, Profenophos, Prothiofos, Quinalphos.	<u>Résultats</u> < LMR* * Limite Maximale de Résidus autorisée

Figure S10 (page 1/4) – Certificate of the chemical analysis for the lot number OF21237 of *Cymbopogon martinii var. motia* (Roxb.) W.Watson (palmarosa) provided by Pranarôm. Screen capture made on 1st September 2020.

Profil CHROMATOGRAPHIQUE

Figure S10 (page 2/4) – Certificate of the chemical analysis for the lot number OF21237 of *Cymbopogon martinii var. motia* (Roxb.) W.Watson (palmarosa) provided by Pranarôm. Screen capture made on 1st September 2020.

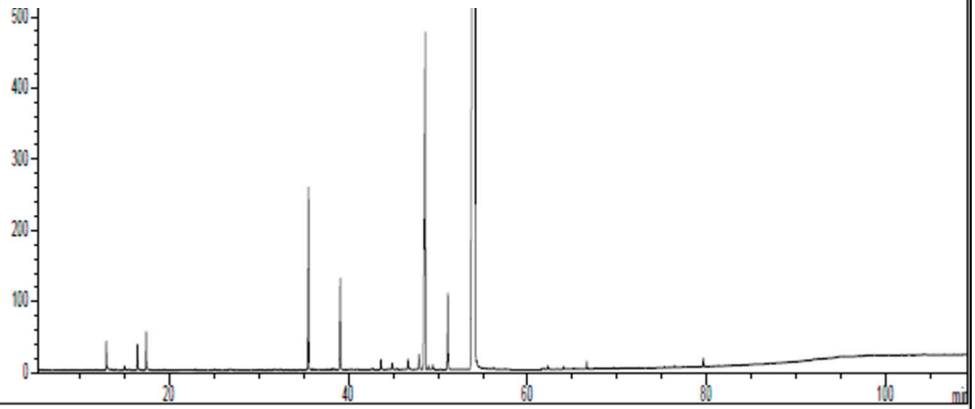

Tableau de résultats 1 : CYMBOPOGON MARTINII

LOT OF21237

Pics	Temps de rétention	Constituants	%
1	5,2	ACETONE	0,01
2	9,6	CAMPHENE	0,01
3	11,0	β-PINENE	0,01
4	11,8	Δ2-CARENE	0,01
5	13,0	**β-MYRCENE**	**0,47**
6	13,5	α-PHELLANDRENE	0,02
7	14,0	o-CYMENE	0,01
8	15,0	**LIMONENE**	**0,07**
14	26,7	ALLO-OCIMENE	0,03
15	31,7	CITRONELLAL	0,02
16	32,4	α-COPAENE	0,02
17	34,7	PLINOL ISOMERE	0,01
18	35,5	**LINALOL**	**2,89**
19	40,1	β1-CUBEBENE	0,01
20	40,6	1-OCTANOL	0,02
21	40,7	ACETATE DE LINALYLE	0,02
22	41,9	SESQUITERPENE	0,01
23	42,5	ISOCARYOPHYLLENE	0,01
24	42,6	ESTER ALIPHATIQUE	0,01
25	38,5	β-ELEMENE	0,02
26	38,2	PLINOL ISOMERE	0,03
27	39,1	β-CARYOPHYLLENE	1,75
28	40,4	SESQUITERPENE	0,01
29	40,9	SESQUITERPENE	0,01
30	41,0	GUAIADIENE ISOMERE	0,01
31	42,6	SESQUITERPENE	0,02
32	42,7	SESQUITERPENE	0,02
33	43,6	α-HUMULENE	0,20
34	44,9	NERAL	0,13
35	45,4	SESQUITERPENE	0,01
36	45,8	γ-MUUROLENE	0,02
37	46,1	FORMIATE DE GERANYLE	0,01
38	46,3	GERMACRENE D	0,01
39	46,6	ACETATE DE NERYLE	0,23
40	46,8	VALENCENE	0,02
41	47,4	β-CURCUMENE	0,01
42	47,9	GERANIAL	0,31
43	48,6	**ACETATE DE GERANYLE**	**8,49**
44	48,7	E,E-α-FARNESENE	0,01
45	48,9	CITRONELLOL	0,08

Figure S10 (page 3/4) – Certificate of the chemical analysis for the lot number OF21237 of *Cymbopogon martinii var. motia* (Roxb.) W.Watson (palmarosa) provided by Pranarôm. Screen capture made on 1st September 2020.

Tableau de résultats 2 : CYMBOPOGON MARTINII

LOT OF21237

Pics	Temps de rétention	Constituants	%
46	49,4	Cis-α-BISABOLENE	0,10
47	51,1	NEROL	1,33
48	54,2	**GERANIOL**	**81,92**
49	54,3	ISOGERANIOL	0,04
50	56,2	BUTYRATE DE GERANYLE	0,02
51	57,5	METHYLBUTYRATE DE GERANYLE	0,01
52	51,6	ALCOOL TERPENIQUE Mw=152	0,01
53	61,7	OXYDE D'ISOCARYOPHYLLENE	0,02
54	61,9	Z-NEROLIDOL	0,01
55	62,3	OXYDE DE CARYOPHYLLENE	0,07
56	64,0	E-NEROLIDOL	0,04
57	65,1	EPOXY-6,7-HUMULENE	0,01
58	66,6	CAPROATE DE GERANYLE	0,12
59	67,7	ESTER GERANIQUE	0,01
60	69,6	COMPOSE ALIPHATIQUE	0,02
61	75,3	ACETATE DE FARNESYLE	0,01
62	76,4	ESTER GERANIQUE	0,03
63	79,6	FARNESOL	0,15
		**TOTAL**	**99,99**

Date de l'analyse – date of the analysis : Mars 2015,

Pranarôm Int.

Figure S10 (page 4/4) – Certificate of the chemical analysis for the lot number OF21237 of *Cymbopogon martinii var. motia* (Roxb.) W.Watson (palmarosa) provided by Pranarôm. Screen capture made on 1st September 2020.
